# Supplementary material for: Multi-species atlas resolves an axolotl limb development and regeneration paradox
Source: Nat Commun. 2023 Oct 10;14:6346. doi: 10.1038/s41467-023-41944-w (PMC10564727; doi:10.1038/s41467-023-41944-w)
Supplement: Supplementary file 1 — Supplementary Information [file 41467_2023_41944_MOESM1_ESM.pdf]

Multi-species atlas resolves an axolotl limb development and regeneration paradox

Supplementary information

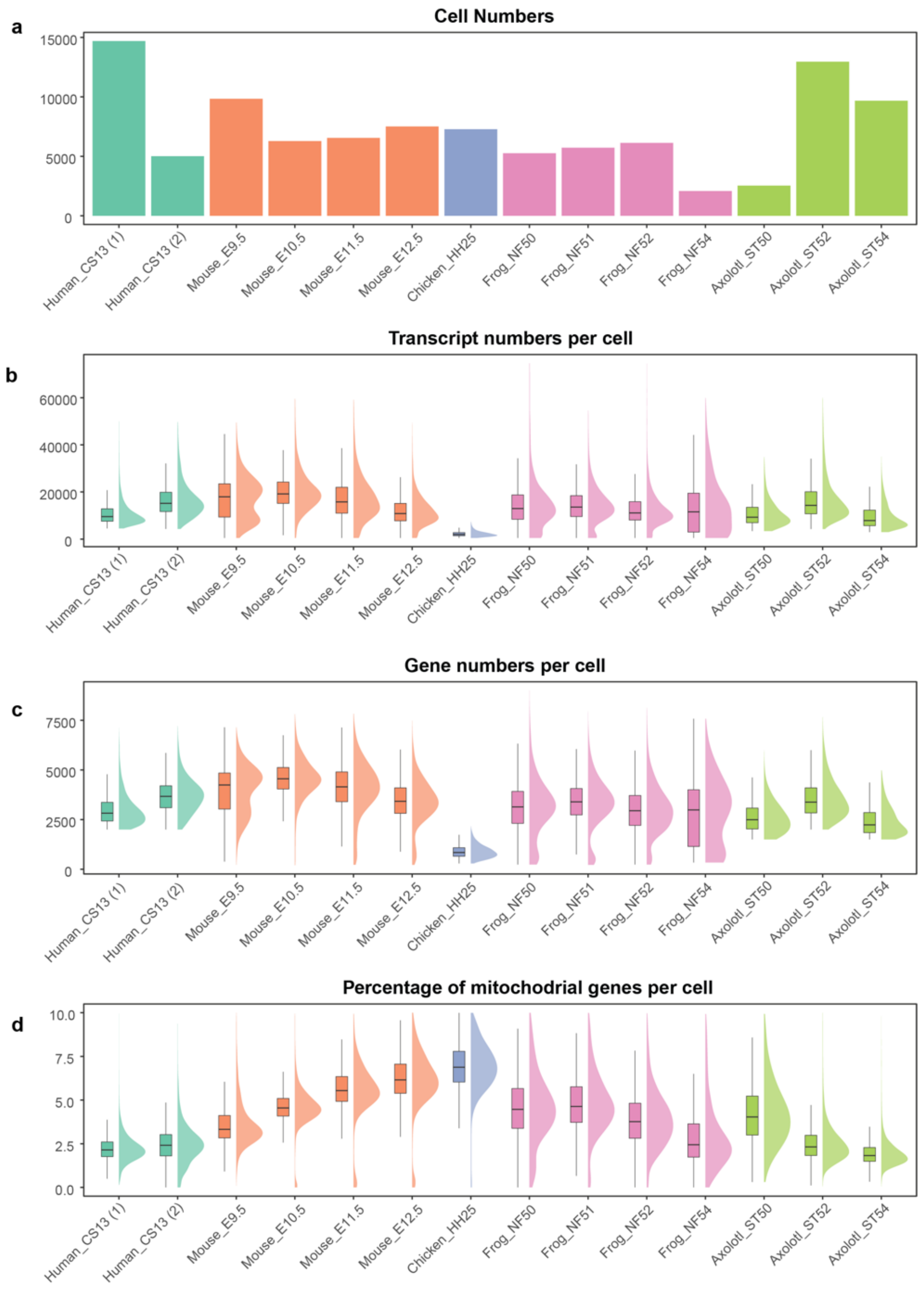

Supplementary Fig. 1 | Quality assessment of the re-analyzed publicly available limb development datasets of five species.

a) Barplot showing the used cell numbers after filtering in each dataset is visualized. Two human datasets of the same developmental stage from independent studies were used and denoted as Human_CS13 (1) and Human CS13 (2). Please see Supplementary Data 1 for full details.

b) Boxplot showing the transcript number per cell in each dataset is visualized. Please see Supplementary Data 1 for full details.

c) Boxplot showing the gene numbers per cell in each dataset is visualized. Please see Supplementary Data 1 for full details.

d) Boxplot showing the percentage of mitochondrial genes per cell in each dataset is visualized. Please see Supplementary Data 1 for full details.

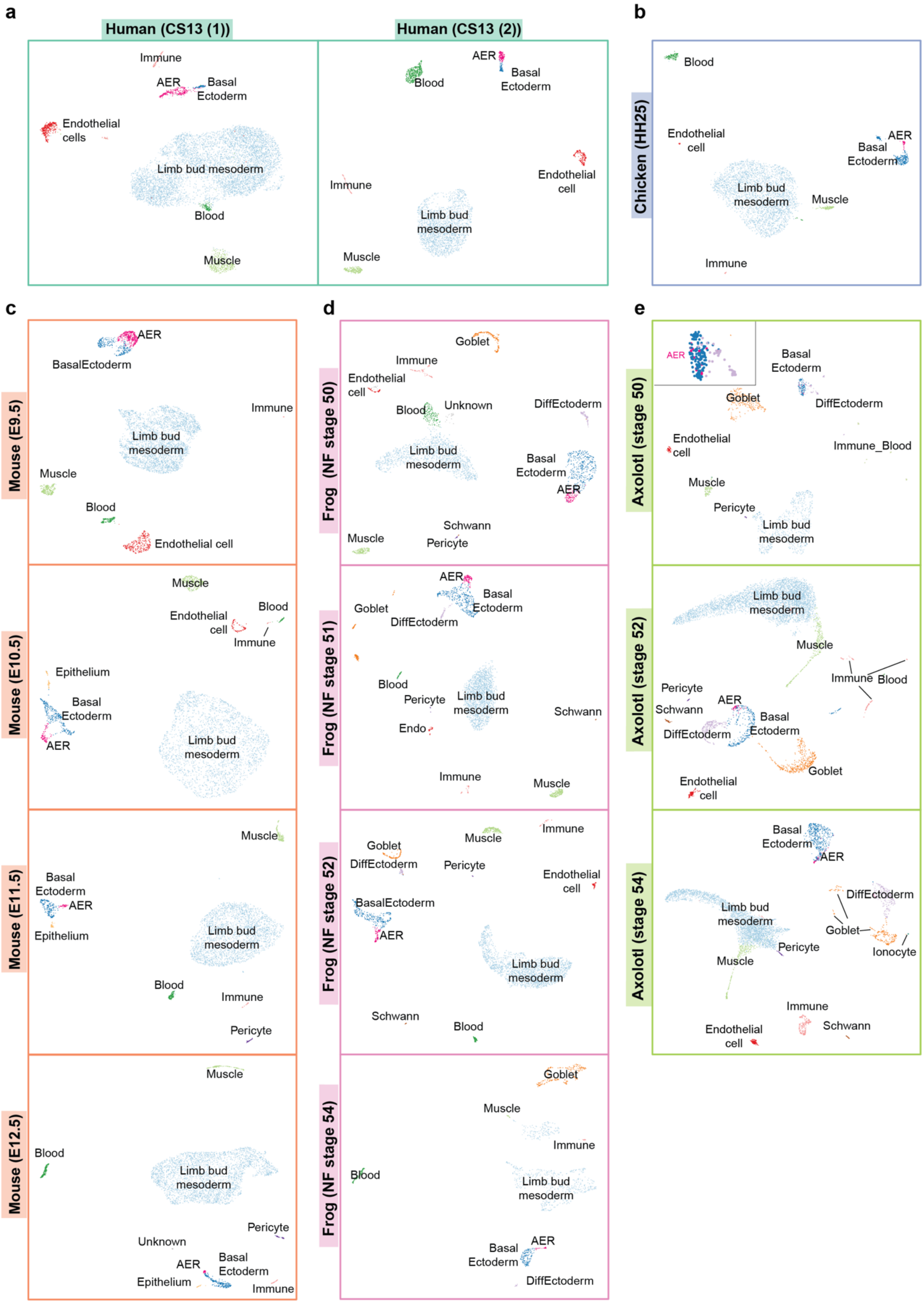

a
Human (CS13 (1))
Immune
AER
Basal Ectoderm
Endothelial cells
Limb bud mesoderm
Blood
Muscle
Human (CS13 (2))
AER
Blood
Basal Ectoderm
Endothelial cell
Immune
Limb bud mesoderm
Muscle
b
Chicken (HH25)
Blood
Endothelial cell
AER
Basal Ectoderm
Limb bud mesoderm
Muscle
Immune
c
Mouse (E9.5)
AER
BasalEctoderm
Immune
Limb bud mesoderm
Muscle
Blood
Endothelial cell
Mouse (E10.5)
Muscle
Blood
Endothelial cell
Immune
Epithelium
Basal Ectoderm
AER
Limb bud mesoderm
Mouse (E11.5)
Muscle
Basal Ectoderm
AER
Epithelium
Limb bud mesoderm
Blood
Immune
Pericyte
Mouse (E12.5)
Muscle
Blood
Limb bud mesoderm
Pericyte
Unknown
Basal Ectoderm
AER
Epithelium
Immune
d
Frog (NF stage 50)
Goblet
Immune
Endothelial cell
Blood
Unknown
DiffEctoderm
Limb bud mesoderm
Basal Ectoderm
AER
Schwann
Muscle
Pericyte
Frog (NF stage 51)
AER
Basal Ectoderm
Goblet
DiffEctoderm
Blood
Pericyte
Limb bud mesoderm
Schwann
Endo
Immune
Muscle
Frog (NF stage 52)
Goblet
Muscle
Immune
DiffEctoderm
Pericyte
Endothelial cell
BasalEctoderm
AER
Limb bud mesoderm
Schwann
Blood
Frog (NF stage 54)
Goblet
Muscle
Immune
Blood
Limb bud mesoderm
Basal Ectoderm
AER
DiffEctoderm
e
Axolotl (stage 50)
AER
Basal Ectoderm
Goblet
DiffEctoderm
Endothelial cell
Immune_Blood
Muscle
Pericyte
Limb bud mesoderm
Axolotl (stage 52)
Limb bud mesoderm
Muscle
Immune
Blood
Pericyte
Schwann
AER
Basal Ectoderm
DiffEctoderm
Goblet
Endothelial cell
Axolotl (stage 54)
Basal Ectoderm
AER
DiffEctoderm
Limb bud mesoderm
Goblet
Pericyte
Ionocyte
Muscle
Immune
Schwann
Endothelial cell

Supplementary Fig. 2 | UMAP visualization of the re-analyzed individual datasets of limb development.

UMAP visualization of individual limb development datasets for (a) human, (b) chicken, (c) mouse, (d) frog, and (e) axolotl are visualized. Two human datasets of the same developmental stage from independent studies were used and denoted as Human_CS13 (1) and Human CS13(2). Lineage and cell type annotations are based on marker genes in Supplementary Fig. 3, and labeled by different colors and text, except the axolotl AER cells, which are identified based on Fig. 1d and re-plotted in this figure. Note that to highlight the identified AER-like cells in the axolotl stage 50 sample, a zoom-in view of the ectoderm population is shown in the upper left corner.

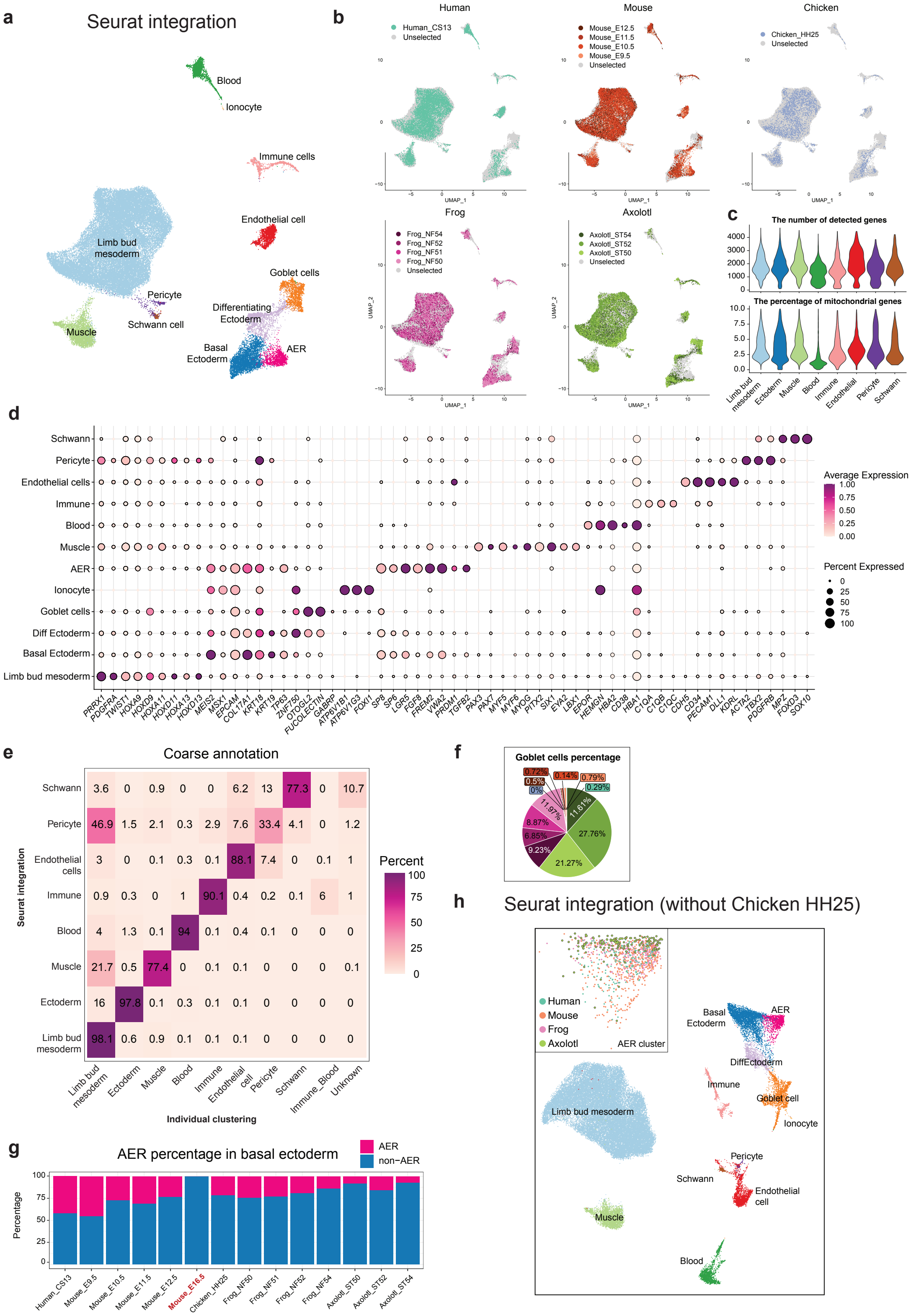
a
Seurat integration
Blood
Ionocyte
Immune cells
Endothelial cell
Limb bud mesoderm
Goblet cells
Pericyte
Schwann cell
Differentiating Ectoderm
Muscle
Basal Ectoderm
AER
b
Human
Human_CS13
Unselected
Mouse
Mouse_E12.5
Mouse_E11.5
Mouse_E10.5
Mouse_E9.5
Unselected
Chicken
Chicken_HH25
Unselected
Frog
Frog_NF54
Frog_NF52
Frog_NF51
Frog_NF50
Unselected
Axolotl
Axolotl_ST54
Axolotl_ST52
Axolotl_ST50
Unselected
UMAP_1
UMAP_2
c
The number of detected genes
The percentage of mitochondrial genes
Limb bud mesoderm
Ectoderm
Muscle
Blood
Immune
Endothelial
Pericyte
Schwann
d
Schwann
Pericyte
Endothelial cells
Immune
Blood
Muscle
AER
Ionocyte
Goblet cells
Diff Ectoderm
Basal Ectoderm
Limb bud mesoderm
PRRX1 PDGFRA TWIST1 HOXA9 HOXD9 HOXA11 HOXD11 HOXA13 HOXD13 MEIS2 MSX1 EPCAM COL17A1 KRT18 KRT19 TP63 ZNF750 OTOGL2 FUCOLECTIN GABRP ATP6V1B1 ATP6V1G3 FOXI1 SP8 SP6 LGR5 FGF8 FREM2 VWA2 PRDM1 TGFB2 PAX3 PAX7 MYF5 MYF6 MYOG PITX2 SIX1 EYA2 LBX1 EPOR HEMGN HBA2 CD38 HBA1 C1QA C1QB C1QC CDH5 CD34 PECAM1 DLL1 KDRL ACTA2 TBX2 PDGFRB MPZ FOXD3 SOX10
Average Expression
Percent Expressed
e
Coarse annotation
Seurat integration
Individual clustering
Percent
Schwann
Pericyte
Endothelial cells
Immune
Blood
Muscle
Ectoderm
Limb bud mesoderm
Limb bud mesoderm
Ectoderm
Muscle
Blood
Immune
Endothelial cell
Pericyte
Schwann
Immune_Blood
Unknown
f
Goblet cells percentage
g
AER percentage in basal ectoderm
AER
non−AER
Percentage
Human_CS13
Mouse_E9.5
Mouse_E10.5
Mouse_E11.5
Mouse_E12.5
Mouse_E16.5
Chicken_HH25
Frog_NF50
Frog_NF51
Frog_NF52
Frog_NF54
Axolotl_ST50
Axolotl_ST52
Axolotl_ST54
h
Seurat integration (without Chicken HH25)
Human
Mouse
Frog
Axolotl
AER cluster
Basal Ectoderm
AER
DiffEctoderm
Immune
Goblet cell
Ionocyte
Limb bud mesoderm
Pericyte
Schwann
Endothelial cell
Muscle
Blood

Supplementary Fig. 3 | Seurat-integrated multi-species limb atlas.

a) UMAP plot of Seurat-integrated multi-species limb atlas is shown. Individual datasets from each species and developmental stage (full list in Supplementary Fig. 2 and Supplementary Data 1) are integrated. Cell lineage and cell type identities are labeled by different colors and text. Please note that this plot is the same as in Fig. 1b.
b) UMAP plots of species and developmental stage contribution to the Seurat-integrated multi-species limb atlas are shown for each species. Species are color-coded and developmental stages are indicated with a shading of the same color.
c) Violin plots of the number of detected genes (top) and the percentage of expressed mitochondrial genes (bottom) in each cell type in the Seurat-integrated multi-species limb atlas. Cell types were defined in Fig. 1a.
d) Dotplot showing used marker genes to annotate clusters. The dot color indicates the mean expression that was normalized to the max of each cell type and to the max of each gene; the dot size represents the percentage of cells with non-zero expressions.
e) A confusion matrix is plotted to determine the annotation accuracy for cells between the Seurat-integrated multi-species atlas and individual maps from Supplementary Fig. 2.
f) Pie chart showing the percentage of cells from each species and developmental stages in the Goblet cells cluster in the Seurat-integrated UMAP in Fig. 1a.
g) Barplot showing the ratio of identified AER cluster to the whole basal ectoderm cluster in indicated species and developmental stages.
h) UMAP plot of Seurat-integrated multi-species limb atlas without the chicken dataset. Cells are colored by their lineages and cell type identities. Inserted box: the species contribution to the AER cluster is visualized. Cells from different species are color-coded.

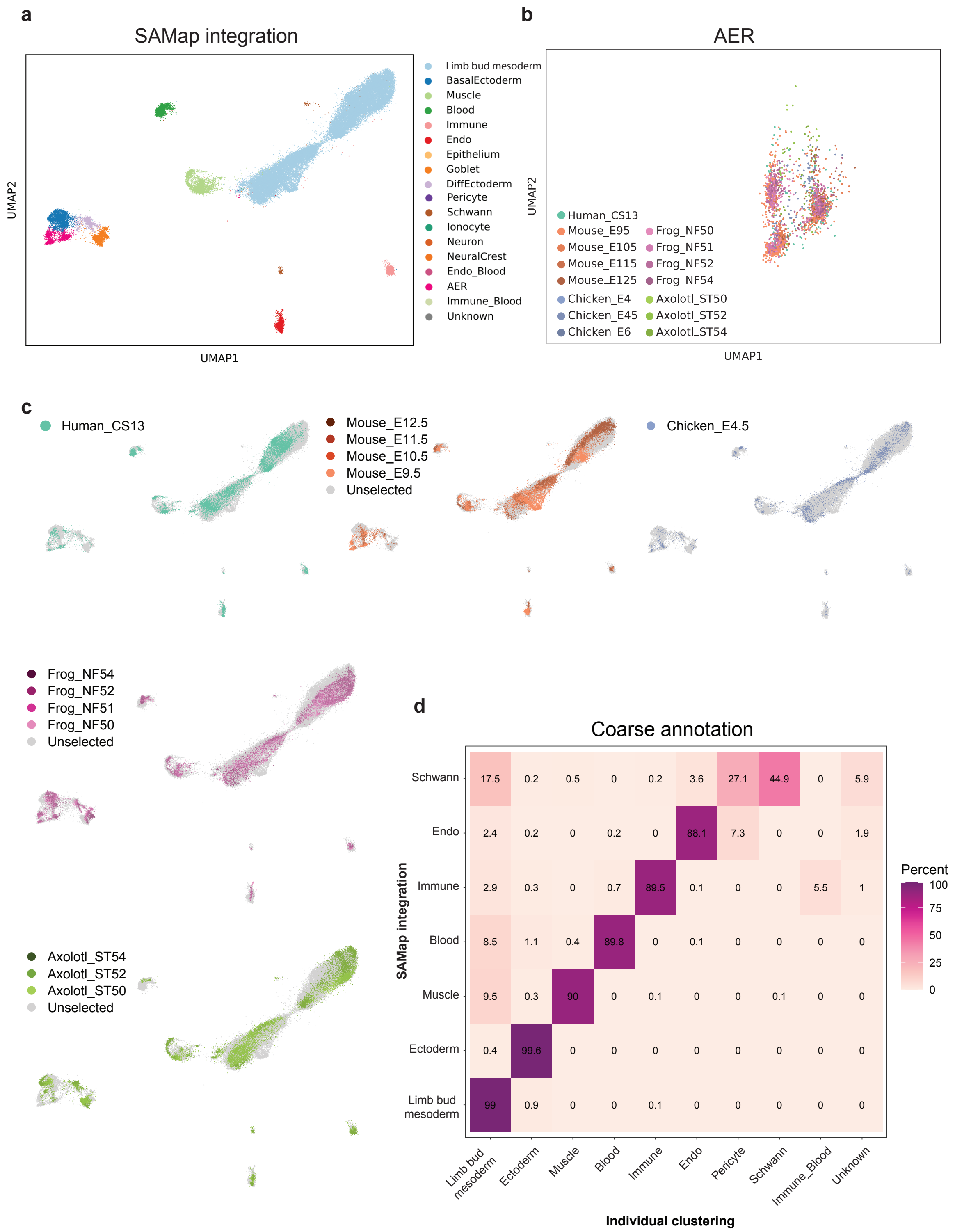

Supplementary Fig. 4 | SAMap-integrated multi-species limb atlas.

a) UMAP plot of SAMap -integrated multi-species limb atlas is shown. Individual datasets from each species and developmental stage (full list in Supplementary Fig. 2 and Supplementary Data 1) are integrated. Clustering and annotation based on marker gene expressions (please see Supplementary Fig. 3) are indicated lineage and cell type identities, and labeled by different colors and text.

b) UMAP plots of species and developmental stage contribution to the SAMap-integrated multi-species limb atlas are shown for each species. Species are color-coded and developmental stages are indicated with a shading of the same color.
c) UMAP plot of species contribution to the SAMap-integrated multi-species limb atlas AER cluster is visualized. Cells from different species are color-coded and the developmental stage is indicated.
d) A confusion matrix is plotted to determine the annotation accuracy between the SAMap-integrated multi-species atlas and individual maps from Supplementary Fig. 2.

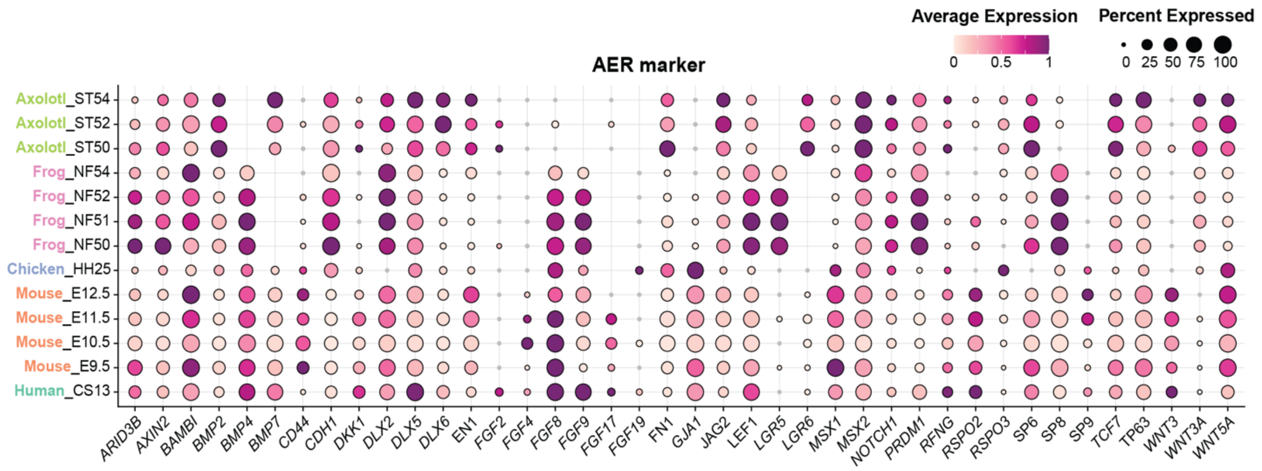

Supplementary Fig. 5 | AER marker expressions in AER cells of the analyzed species. Dotplot showing an extended list of AER markers. The dot color indicates the mean expression that was normalized to the max of each cell type and to the max of each gene; the dot size represents the percentage of cells with non-zero expression. Please note that some of the genes in this figure and their values are the same as in Fig. 1e.

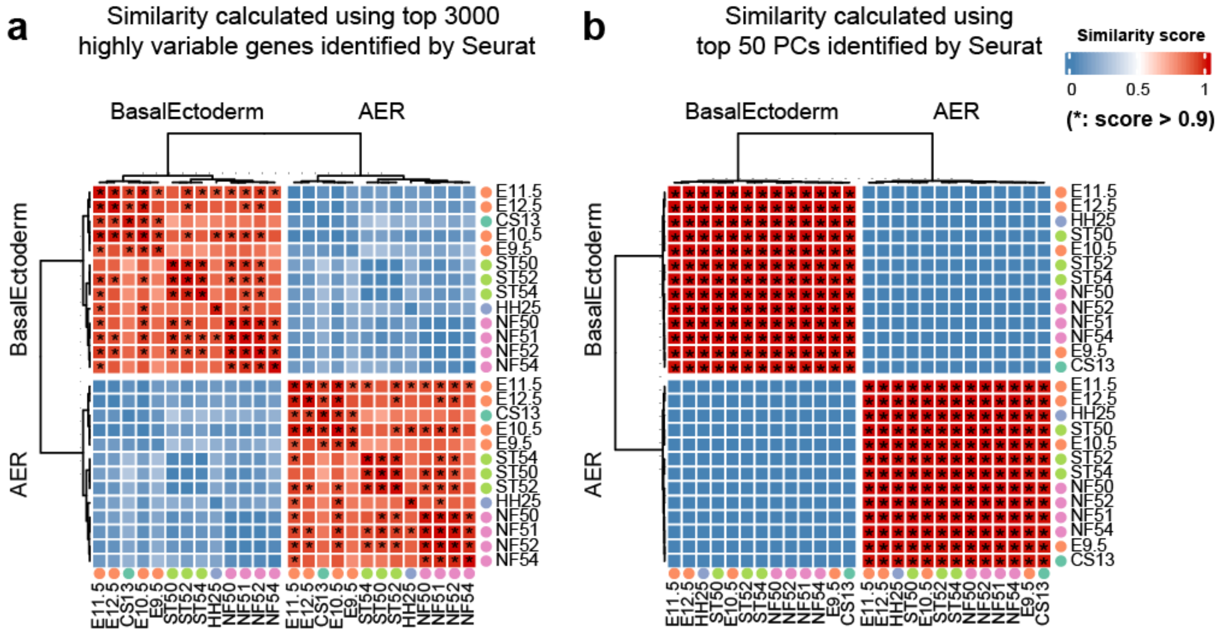

Supplementary Fig. 6 | Basal ectoderm and AER clusters similarities of the analyzed species. Heatmaps showing the pair-wise similarity scores of basal ectoderm and AER clusters across species. X and Y axes indicate different species with their developmental stages. Similarity scores are calculated by using (a) the top 3000 highly variable genes, and (b) the top 50 principal components. Asterisks (*) denote the pairs with similarity scores above 0.9. AER clusters are compared to basal ectoderm clusters to highlight specific transcriptome profiles.

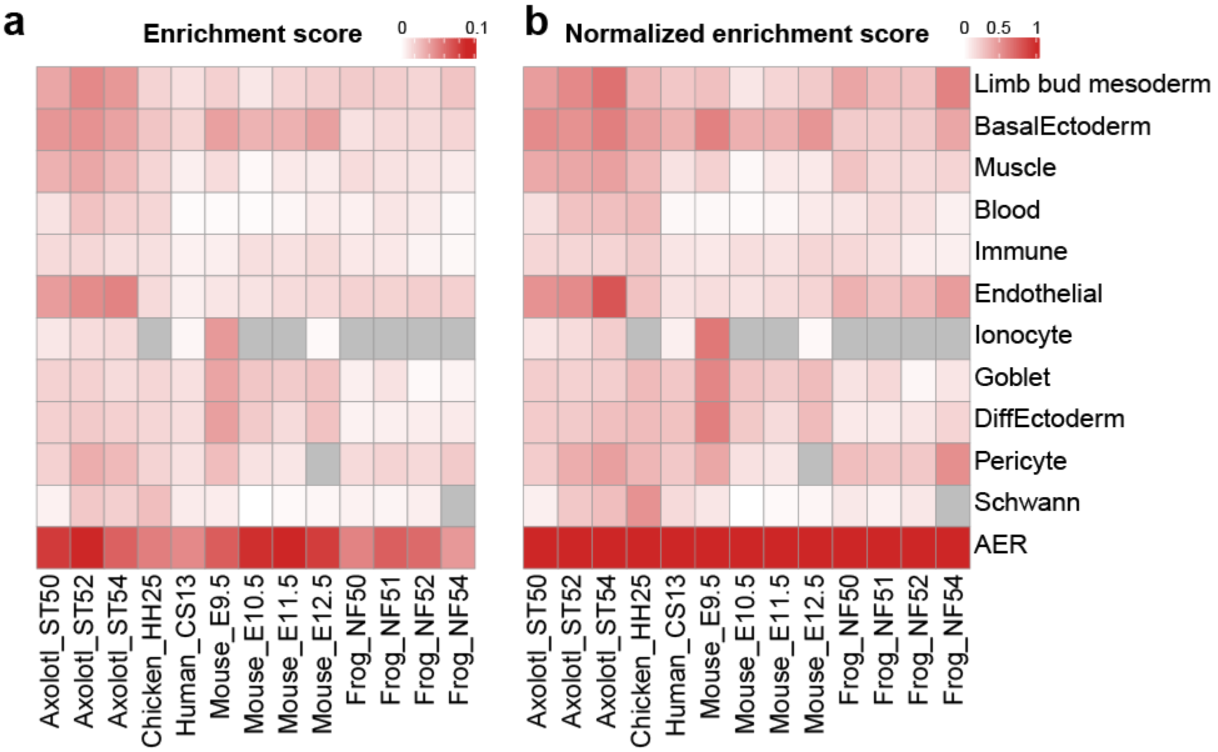

Supplementary Fig. 7 | Signaling ligand gene set enrichment scores for lineages and cell types in the analyzed species.

Heatmap showing the (a) calculated and (b) max-normalized signaling ligand gene set enrichment scores for each annotated population in the multi-species limb atlas (Fig. 1b). Grey indicates missing cell cluster. Please note that the same data for the basal ectoderm and the AER clusters are shown in Fig. 1g.

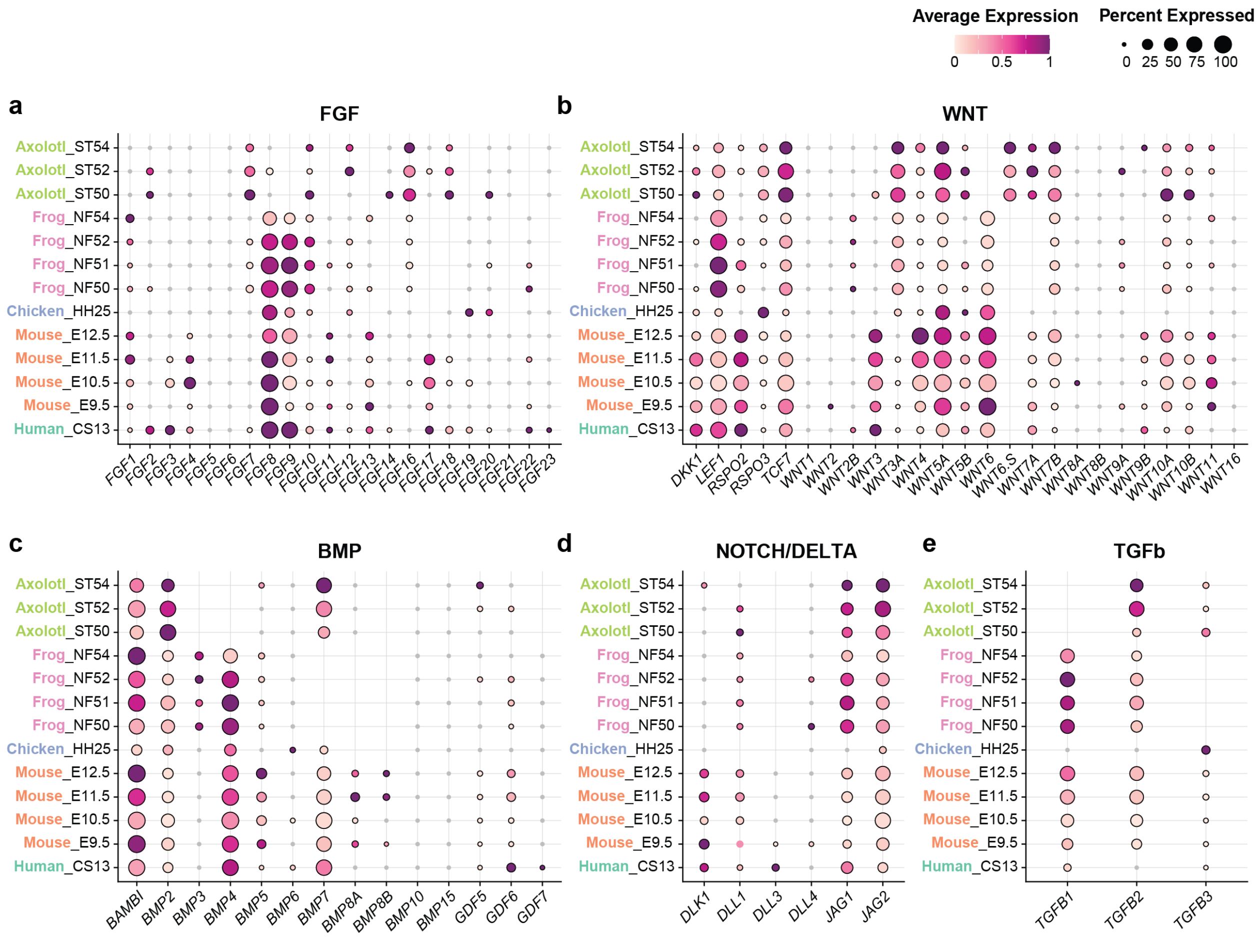

Supplementary Fig. 8 | Signaling pathways-associated gene expressions in AER cells in the analyzed species.

Dotplots showing expression profiles of various genes from FGF (a), WNT (b), BMP (c), NOTCH (d), and TGFb (e) pathways. The dot color indicates the mean expression that was normalized to the max of each cell type and to the max of each gene; the dot size represents the percentage of cells with non-zero expression.

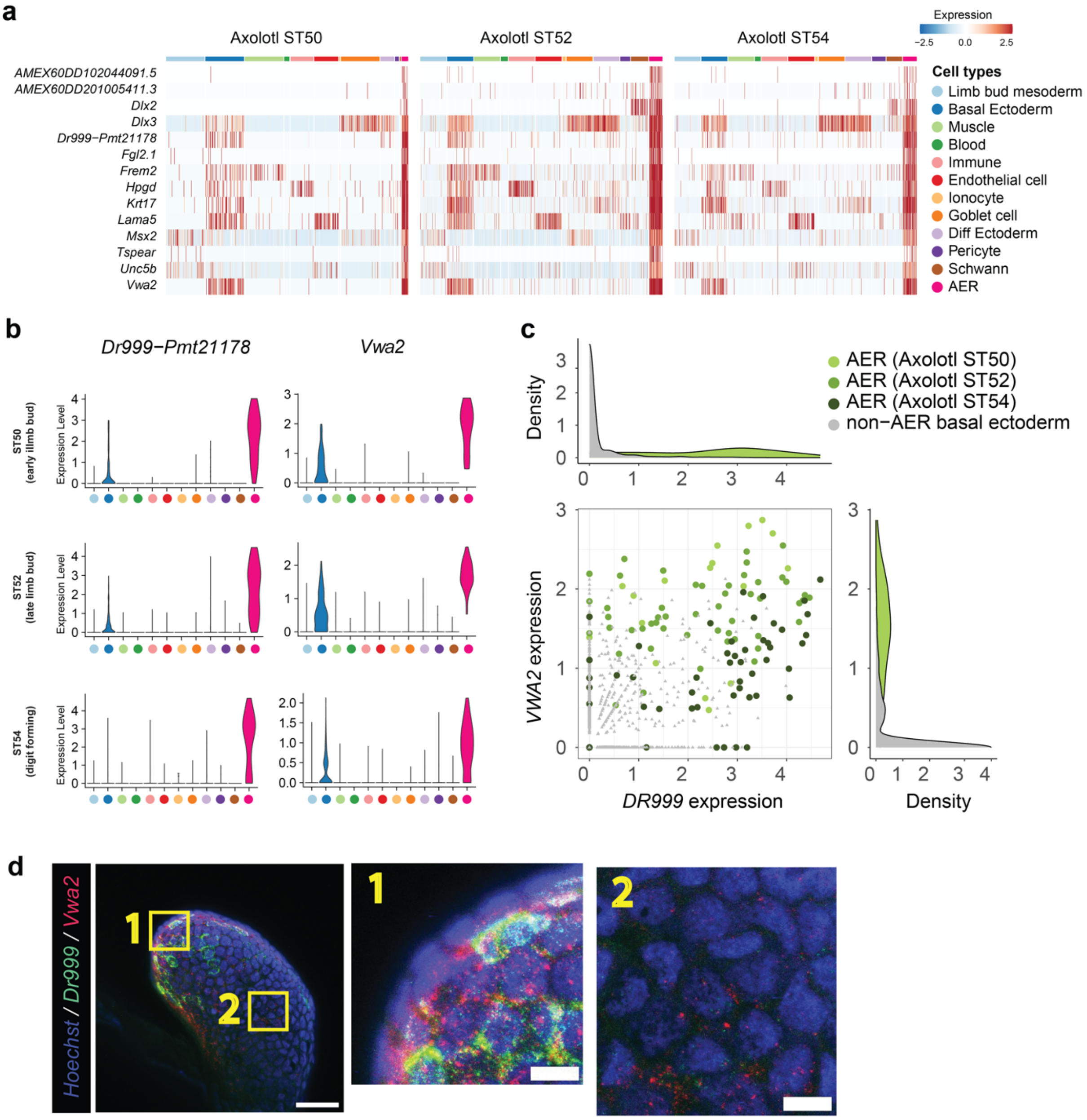

Supplementary Fig. 9 | Spatial organization of axolotl AER cells during limb development.

a) Heatmap showing the expression of 14 shared AER-specific differentially expressed genes in different cell types in axolotl developing limbs. Stages are indicated on the top.

b) Violin plots showing expression levels of the putative axolotl AER markers are shown. Please note that the analyzed scRNA-Seq datasets are for hindlimbs, and morphologies of corresponding developing limbs are indicated in the figure.

c) Co-expression of AER-like genes in the ectoderm in axolotl limb buds. Scatter plot (lower left) shows the co-expression pattern of *Dr999-Pmt21178* and *Vwa2*. Each dot represents a cell whose location is defined by its expression of *Dr999-Pmt21178* (x-axis) and *Vwa2* (y-axis). AER-like cells are colored in shades of green while non-AER ectoderm in grey. Density plots representation of the distribution of the *Dr999-Pmt21178* (top) and *Vwa2* (lower right) expression in AER (in green) and non-AER ectoderm (in grey).

d) (Left) Merged image of the top row in Fig. 2a. Scale bar 100 µm. (Right) Enlarged views of corresponding regions indicated by the numbered yellow boxes. Scale bar 20 µm.

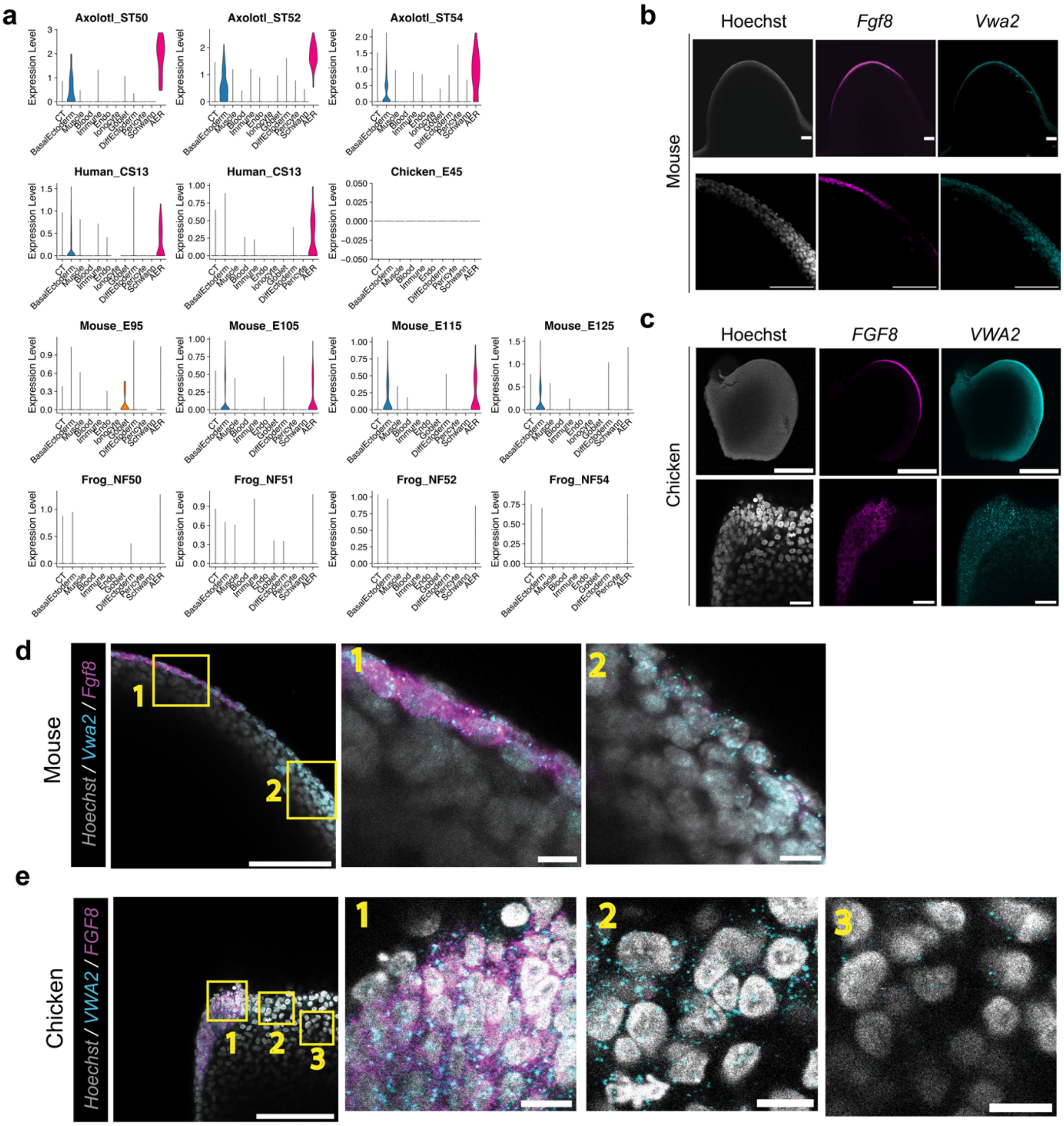

Supplementary Fig. 10 | *Vwa2* expression in humans, mice, chickens, and frogs.

a) Violin plots showing *Vwa2* expression across all cell types in indicated species and developmental stages based on the analyzed scRNA-Seq datasets.

b) Max-projection confocal image of mouse E10.5 hindlimb limb buds stained for *Fgf8*, and *Vwa2* mRNA via HCR. Gray, Hoechst; Magenta, *Fgf8* mRNA; Cyan, *Vwa2* mRNA. Scale bar: 100 µm for both.

c) Max-projection confocal image of chicken HH22 hindlimb buds stained for *Fgf8*, and *Vwa2* mRNA via HCR. Gray, Hoechst; Magenta, *Fgf8* mRNA; Cyan, *Vwa2* mRNA. Scale bars: (Top) 250 µm and (Bottom) 25 µm.

d) Merged image of (b). Zoom-in views of yellow boxes regions are shown by side. Scale bar: 100 µm and 10 µm for enlarged images.

e) Merged image of (c). Zoom-in views of yellow boxes regions are shown by side. Scale bar: 100 µm for full image and 10 µm for enlarged images.

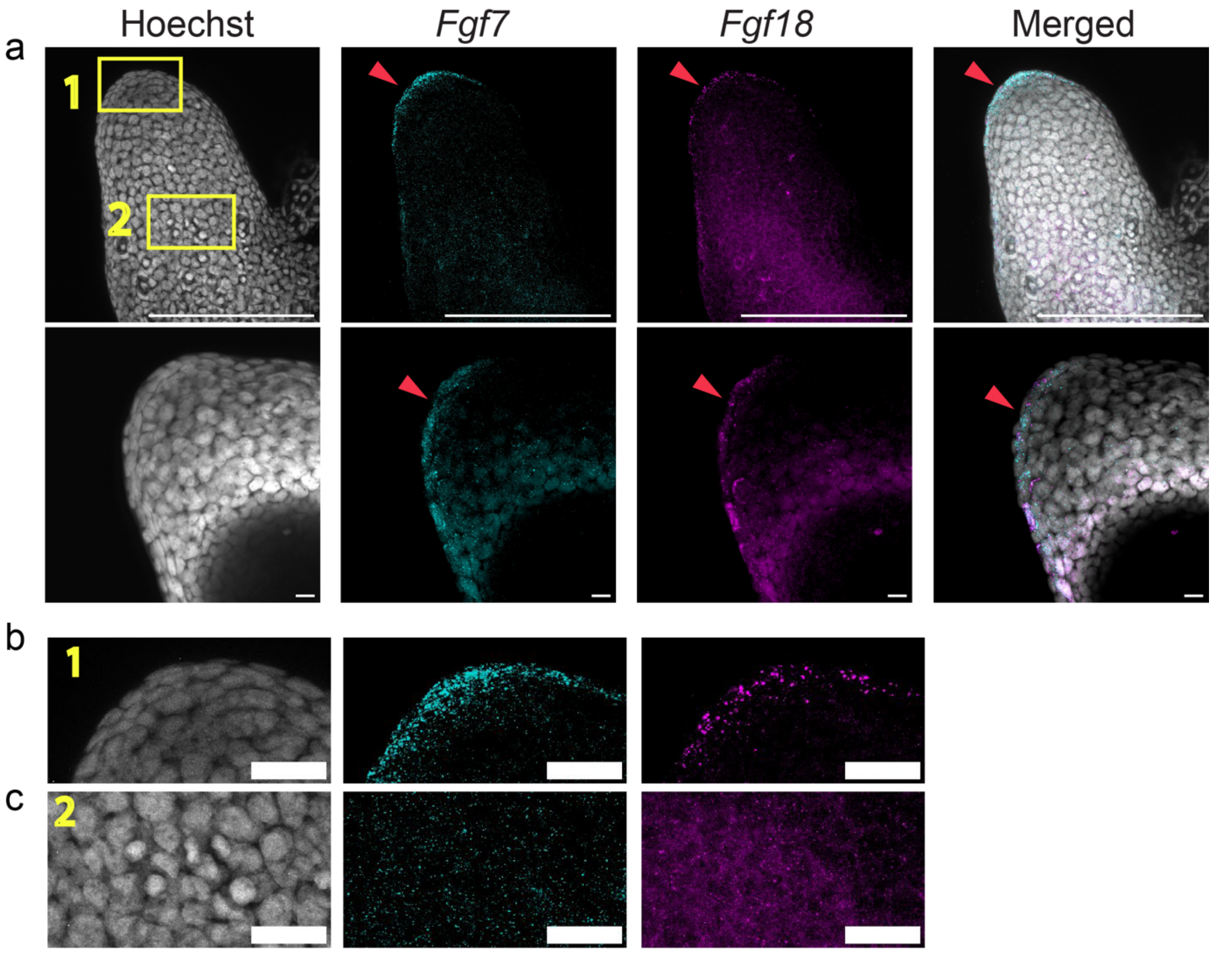

Supplementary Fig. 11 | AER-specific Fgf expression in developing axolotl limbs.

a) Max-projection confocal image of axolotl Stage 53 hindlimb buds stained for *Fgf7* and *Fgf18* mRNA via HCR. Gray, Hoechst; Cyan, *Fgf7*; Magenta, *Fgf18* mRNA; Scale bars: (Top) 100 µm and (Bottom) 20 µm. The red arrow indicates the target signals.

b) Zoomed region of (a) indicated by yellow box number 1 shows the target signals. Scale bars: 20 µm.

c) Zoomed region of (a) indicated by yellow box number 2 shows high background signals. Scale bars: 20 µm.

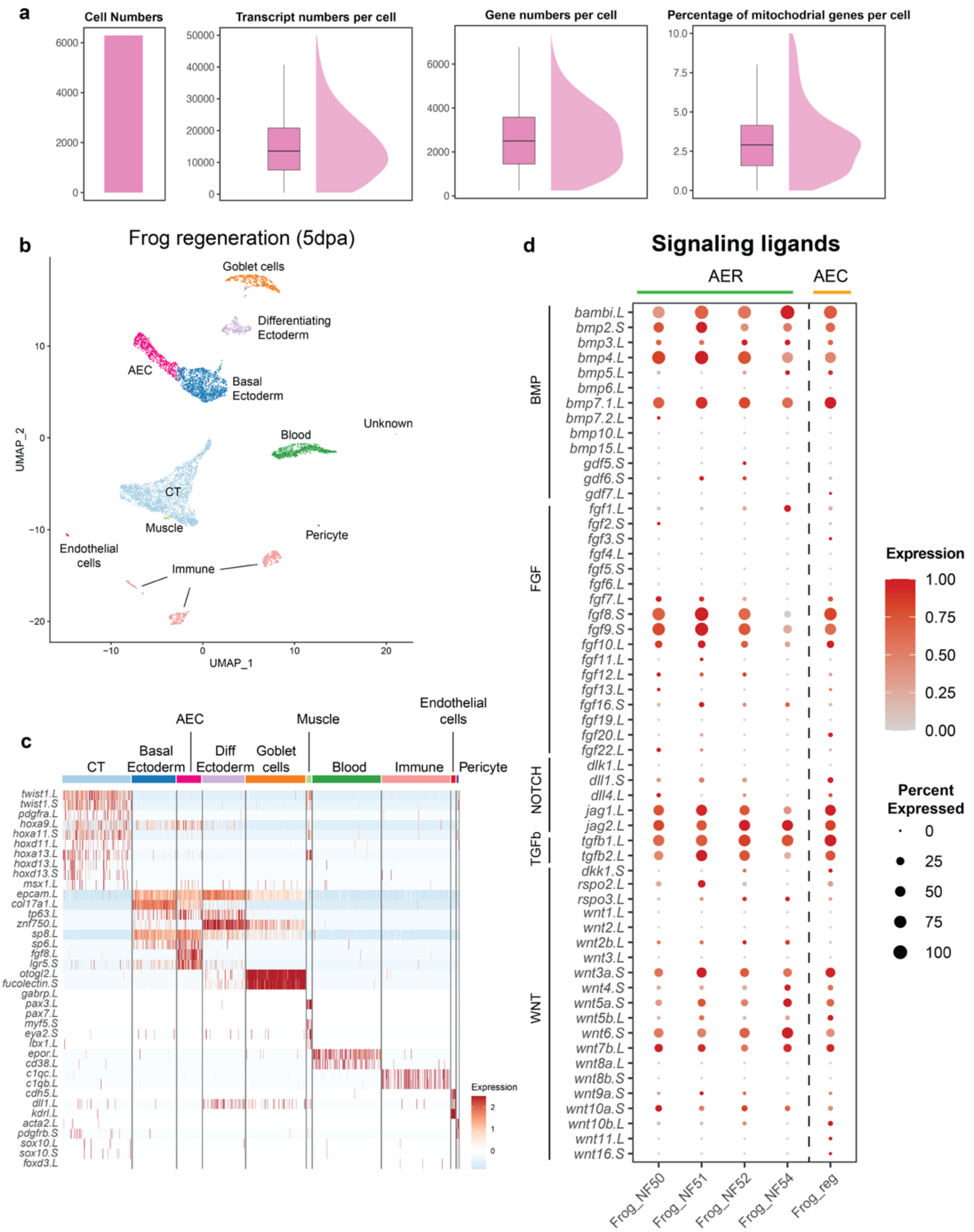

Supplementary Fig. 12 | Quality assessment of the re-analyzed publicly available *Xenopus* limb regeneration dataset.

a) Barplots showing the used cell numbers, the transcript number per cell, the gene numbers per cell, and the percentage of mitochondrial genes per cell after filtering in *Xenopus laevis* 5 days-post amputation (dpa) limb regeneration dataset are visualized.
b) UMAP plot of the identified and annotated clusters. Clusters were annotated based on marker genes listed in Supplementary Fig. 12c.
c) Heatmap showing the expression profile of marker genes that are used to annotate clusters.
d) Dotplot showing signaling ligand expressions in the AER or AEC clusters during *Xenopus laevis* limb development and regeneration, respectively. Please note that a similar figure was generated in Aztekin et al, 2021, but in this figure, NF Stage 50, 51, and 52 from Lin et al. 2021 were used.

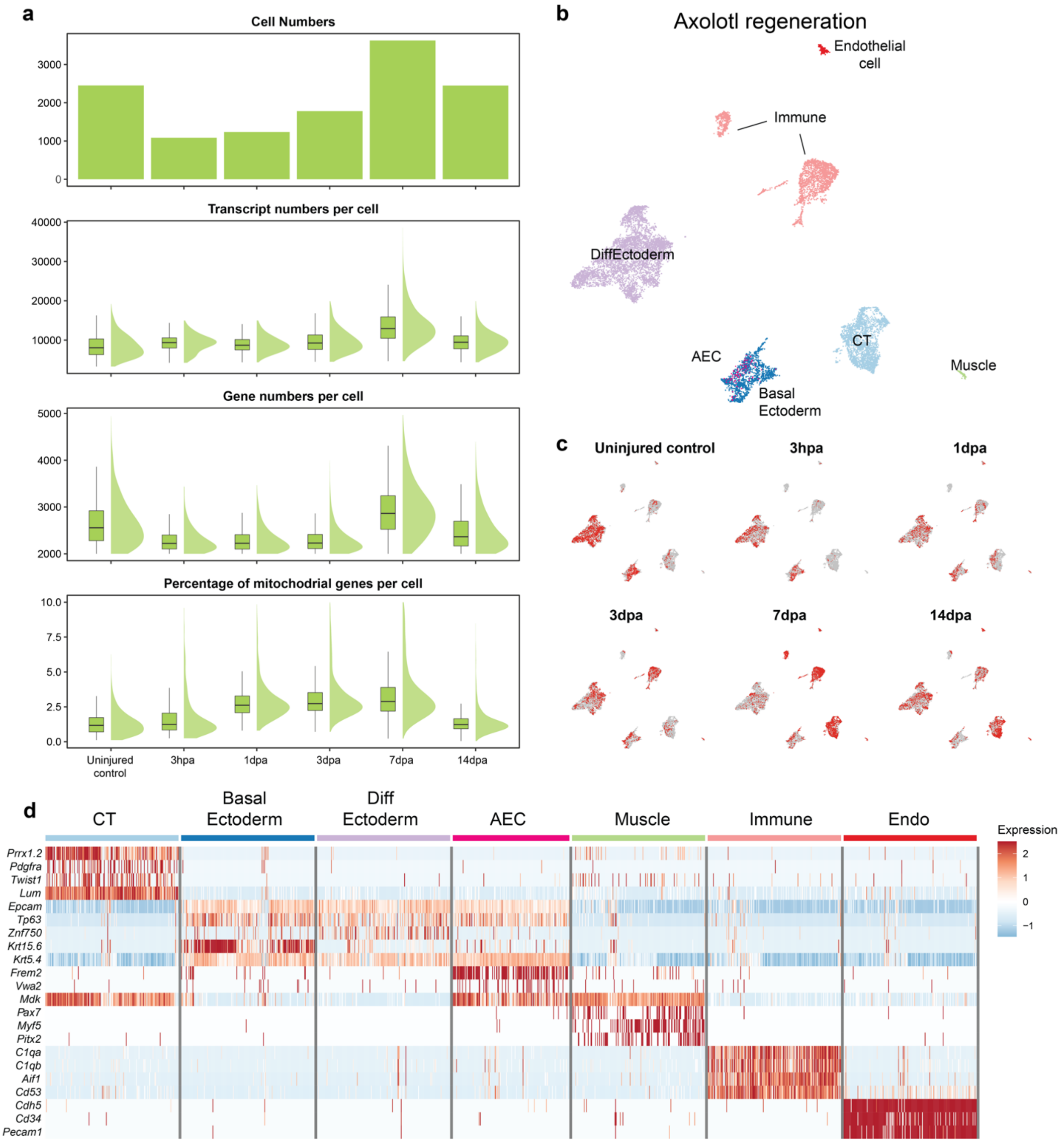

Supplementary Fig. 13 | Quality assessment of re-analyzed publicly available axolotl limb regeneration dataset.

a) Barplots showing the used cell numbers, the transcript number per cell, the gene numbers per cell, and the percentage of mitochondrial genes per cell after filtering in axolotl limb regeneration datasets are visualized.

b) UMAP plot of the identified and annotated clusters. Clusters were annotated based on marker genes listed in Supplementary Fig. 13d. The AEC cells were identified by the analysis described in the text and in Fig. 3a-c, then re-plotted and colored dark pink in this figure.

c) Sample contribution to the axolotl regeneration dataset is visualized. Red dots indicate cells from the selected sample; gray dots indicate the other cells. hpa: hours-post amputation; dpa: days-post amputation.

d) Heatmap showing the expression profile of marker genes that are used to annotate clusters, except AEC cells and their marker expressions indicated following the analysis indicated in text and Fig. 3a-c.

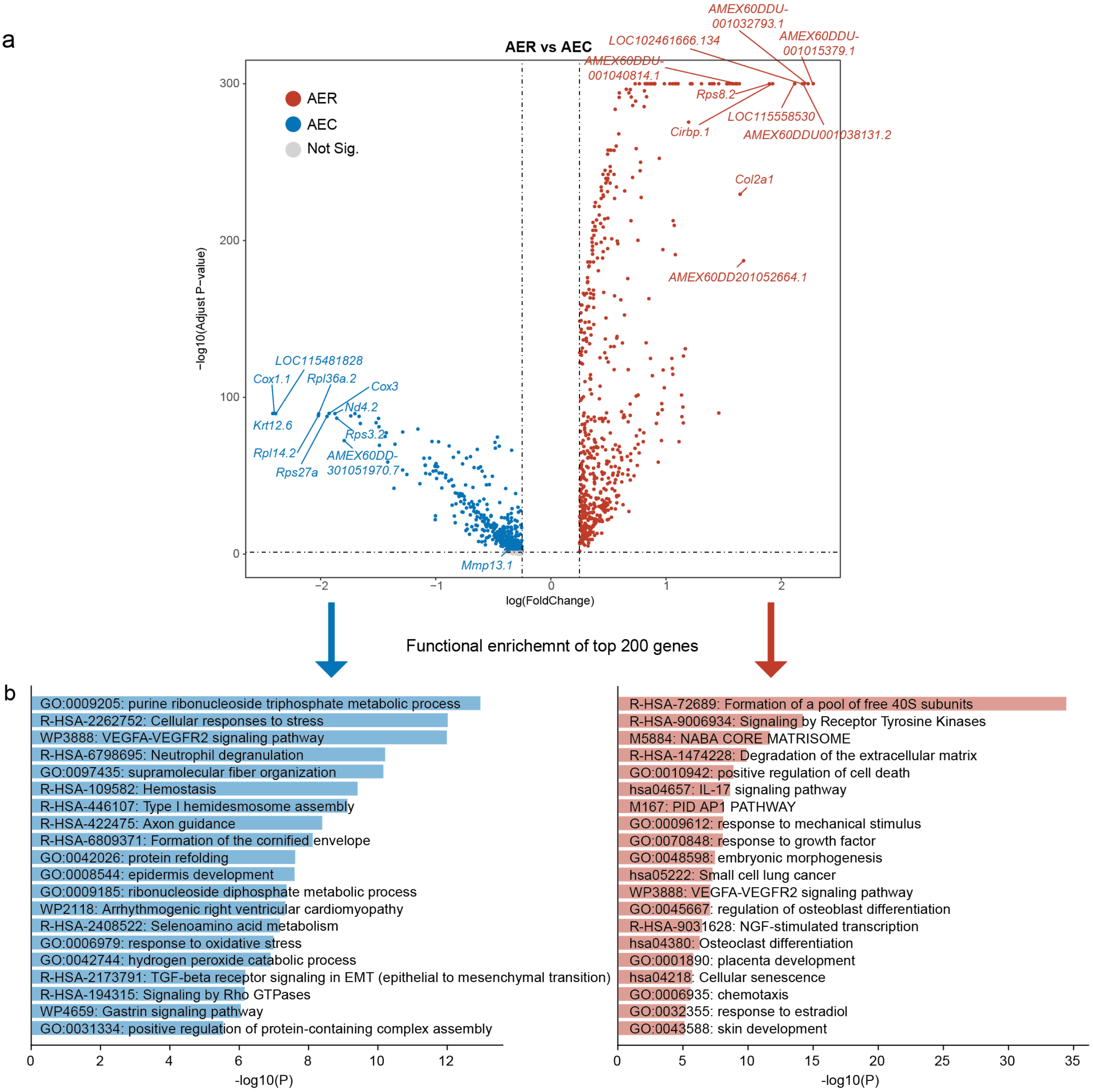

Supplementary Fig. 14 | Differentially expressed gene analysis between axolotl AER and AEC cells.

a) Volcano plot showing the differentially expressed genes (DEGs) between axolotl AER and AEC populations. Red and blue dots indicate genes significantly enriched in the AER or the AEC, respectively. Gray dots indicate statistically not significant genes. The top 10 DEGs are labeled.

b) Barplots showing enriched GO terms based on the top 200 DEGs (ordered by fold change) in AER (red) and AEC (blue) cells.

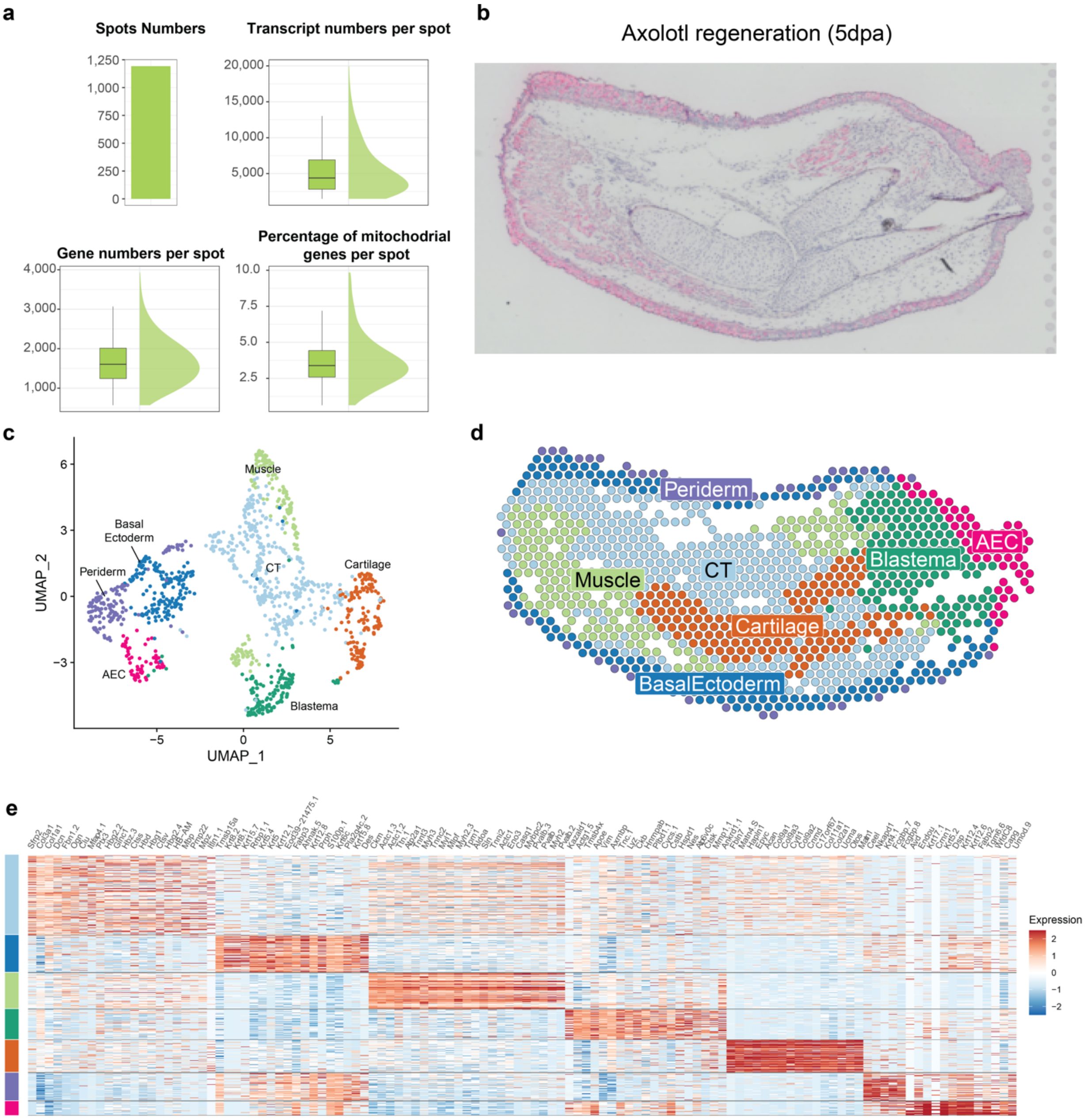

Supplementary Fig. 15 | Quality assessment and clustering results of axolotl 5 days-post-amputation 10X Visium spatial transcriptomics (Visium) dataset.

a) Barplots showing used Visium spot numbers, transcript number per spot, gene numbers per spot, and the percentage of mitochondrial genes per spot after filtering are visualized.

b) 5 dpa axolotl limb regeneration tissue section that is used for the Visium is shown and stained for hematoxylin and eosin. Please note that this image is the same as Fig. 3f but added in this figure for easier visual comparison to Supplementary Fig. 15d, which contains full annotation.

c) UMAP plot of the Visium clusters is shown. Clusters were annotated by marker gene expressions (Supplementary Fig. 15e) and confirmed by morphologies in hematoxylin and eosin staining on the tissue section in Fig. 3f and Supplementary Fig. 15b. The identified clusters are color-coded and tissue annotation is labeled.

d) Visium spots were colored by tissue types identified in Supplementary Fig. 15c.

e) Heatmap showing the top 20 DEGs (ordered by fold change) for the identified clusters is shown.

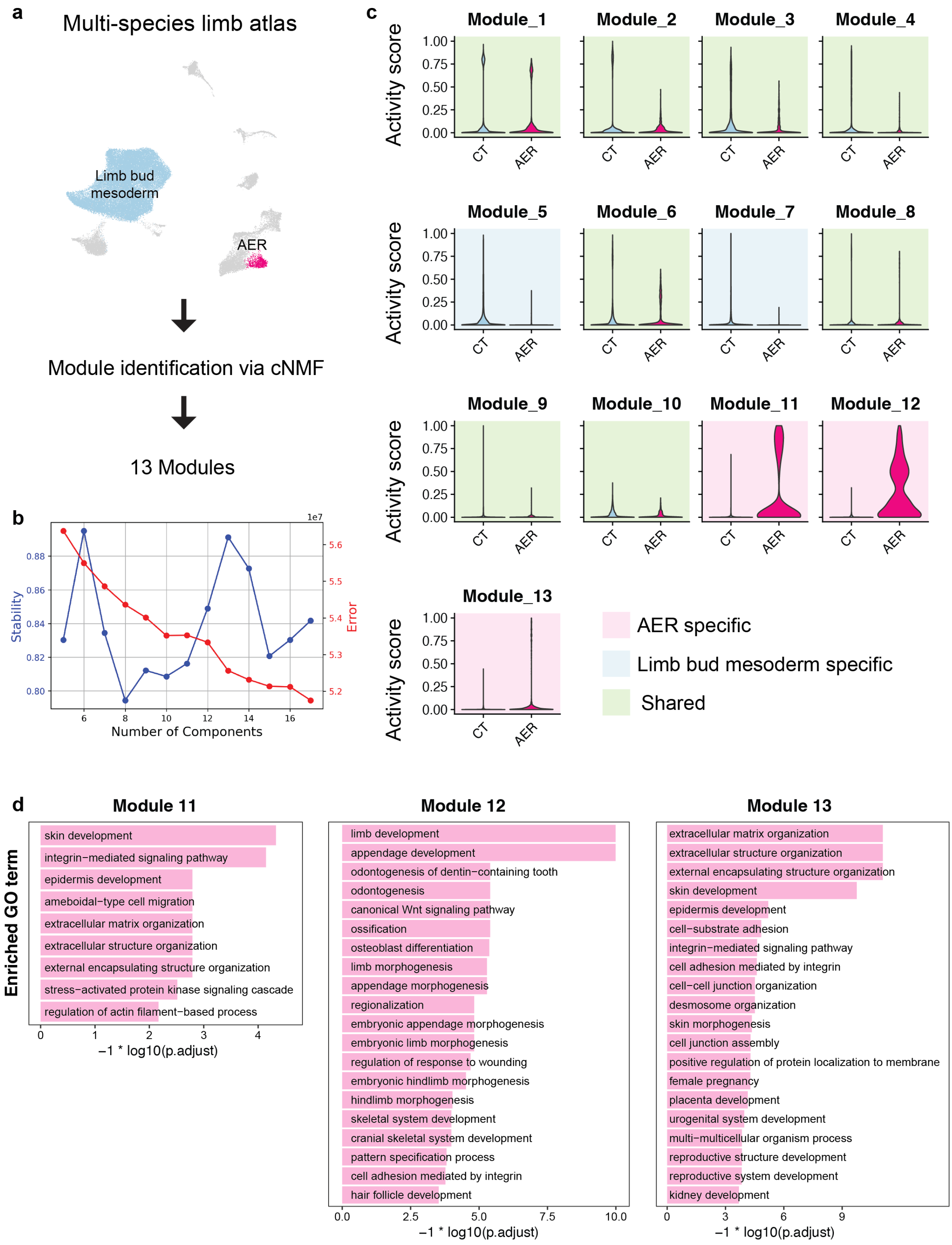

Supplementary Fig. 16 | Consensus nonnegative matrix factorization (cNMF) to identify AER-related modules.

a) The strategy to perform consensus negative matrix factorization (cNMF) is described. cNMF was used on AER and CT clusters to identify cell identity and cell activity modules.

b) Stability (blue line) is measured by the Euclidean distance silhouette score of the clustering and Frobenius error of the consensus solution (red line) for each tested k value (module number) shown in the X axis. Please see Methods for more detail.
c) Violin plots showing activity scores for identified cNMF modules in CT and AER clusters. Modules specific to AER, CT, or shared are color-coded: pink, AER-specific modules; blue, CT-specific modules; green, shared modules.
d) Enriched GO terms are determined for the top 100 genes (ordered by gene expression program scores) in the identified AER-specific modules. Barplot showing the top GO is visualized, and GO terms are labeled.

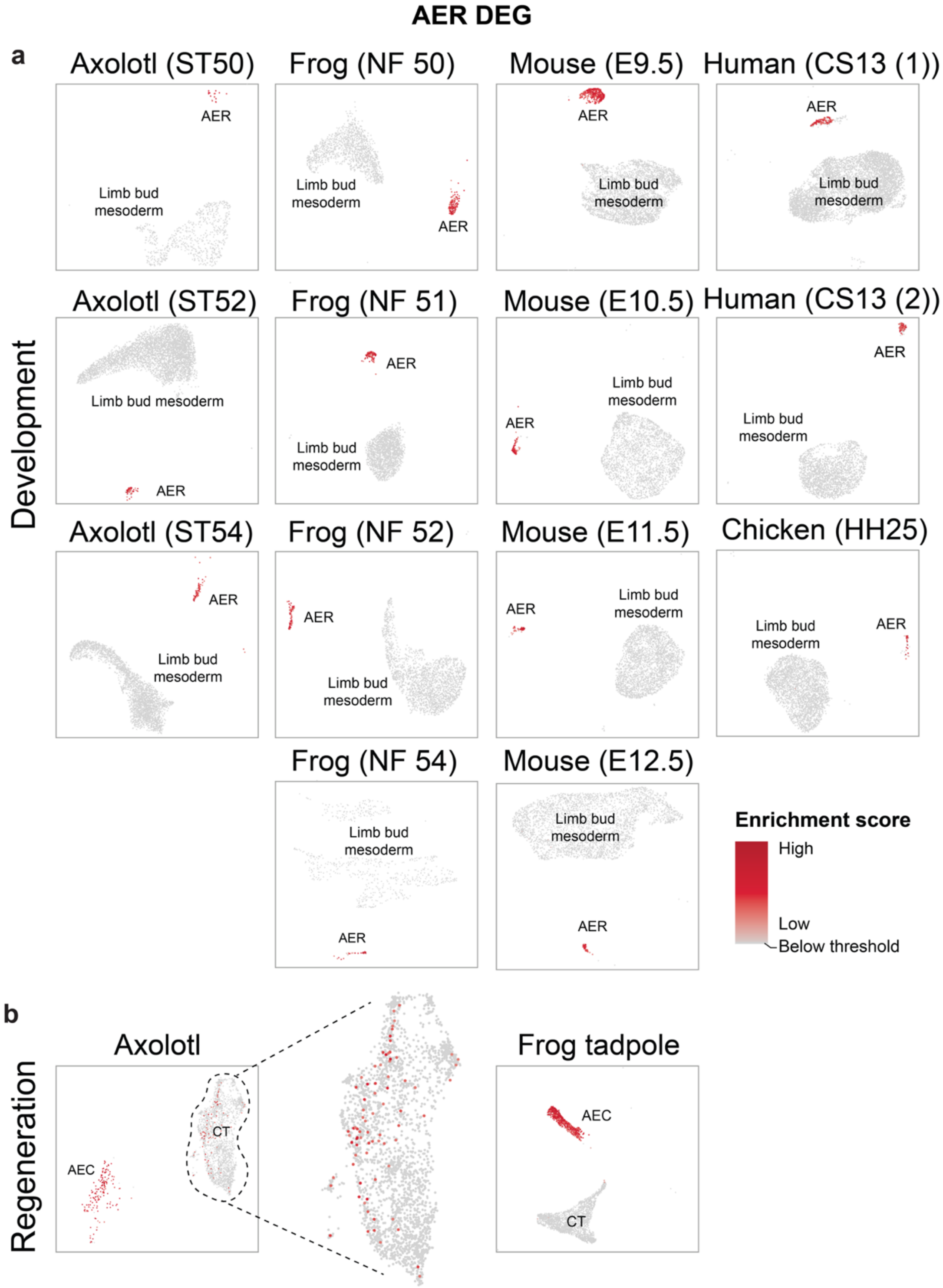

Supplementary Fig. 17 | Single cell gene set enrichment analysis (scGSEA) using differentially expressed AER gene sets for individual datasets.

UMAP visualization of the scGSEA of indicated datasets of limb development (a) and regeneration (b) based on the top 500 AER DEGs in multi-species limb atlas (Fig. 1b). The enrichment scores are indicated in shades of red. Cells failed to pass the enrichment threshold are colored in grey.

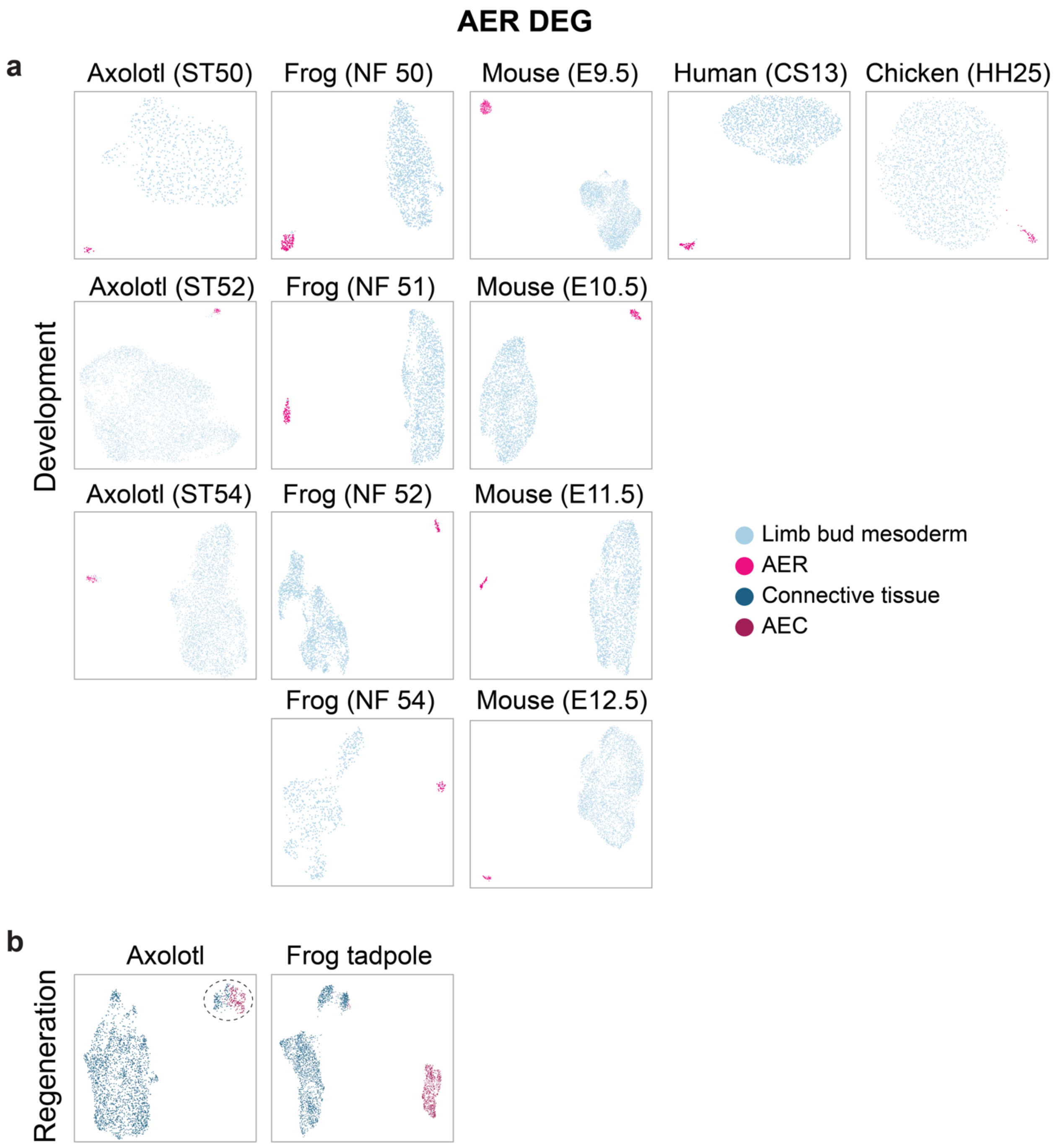

Supplementary Fig. 18 | Clustering based on differentially expressed AER gene sets for individual datasets.

UMAP visualization of the clustering of indicated datasets of limb development (a) and regeneration (b) based on the top 500 AER DEGs in multi-species limb atlas (Fig. 1b) for individual datasets. In development datasets: light blue, limb bud mesoderm cells; pink, AER cells. In regeneration datasets: dark blue, CT cells; dark pink, AEC cells. Connective tissue cells gathered with the AEC population are highlighted with a dashed line. Please note that Mouse E10.5, chicken E4.5, human CS13, frog ST51, and axolotl ST52 development datasets, and the axolotl and frog tadpole regeneration datasets are also shown in Fig 4b and c.

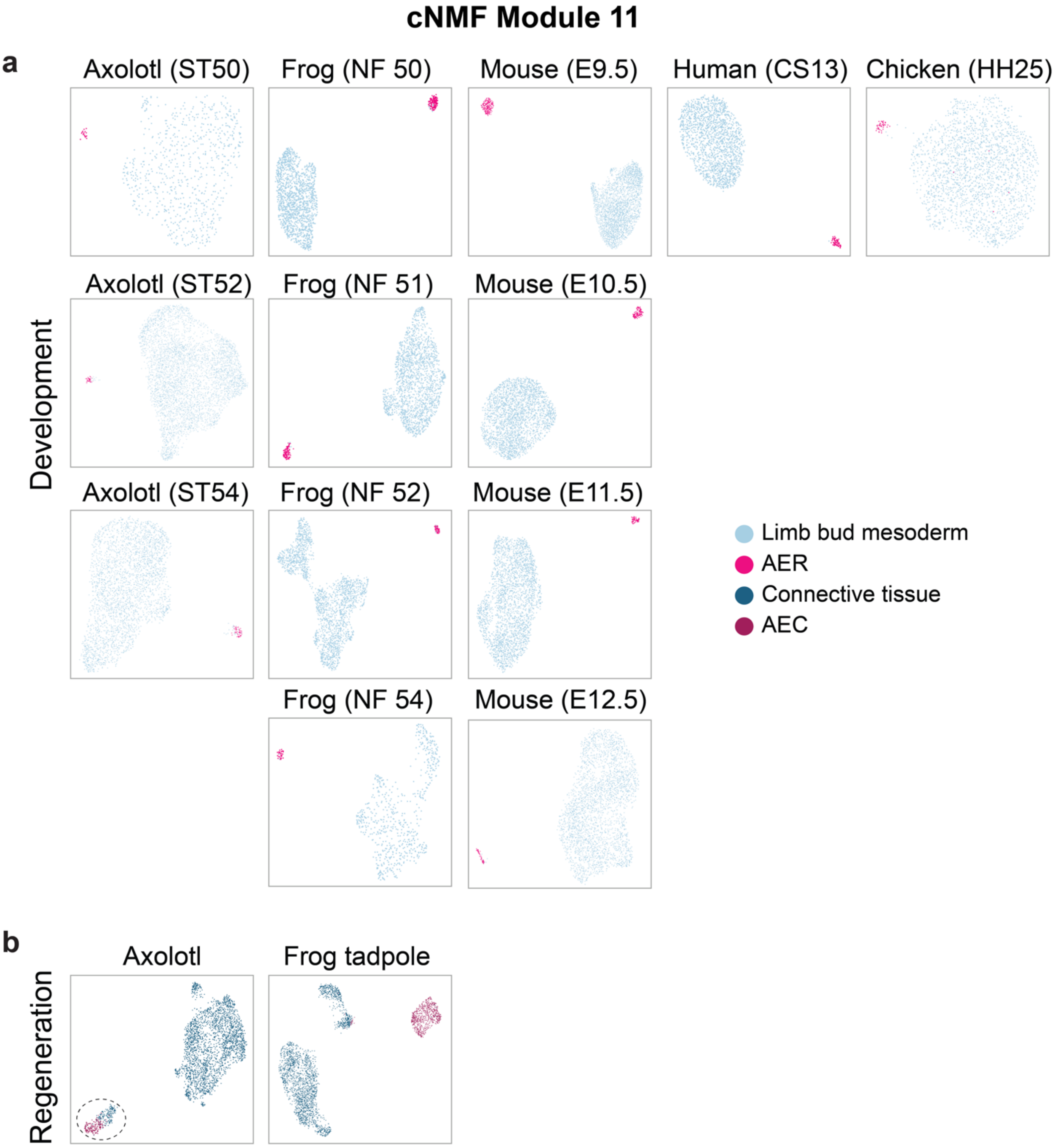

Supplementary Fig. 19 | Clustering based on cNMF module 11 for individual datasets. UMAP visualization of the clustering of indicated datasets of limb development (a) and regeneration (b) based on the top 500 genes from AER-specific cNMF module 11 (Supplementary Fig. 16 and Supplementary Data 4). In development datasets: light blue, limb bud mesoderm cells; pink, AER cells. In regeneration datasets: dark blue, CT cells; dark pink, AEC cells. Connective tissue cells gathered with the AEC population are highlighted with a dashed line.

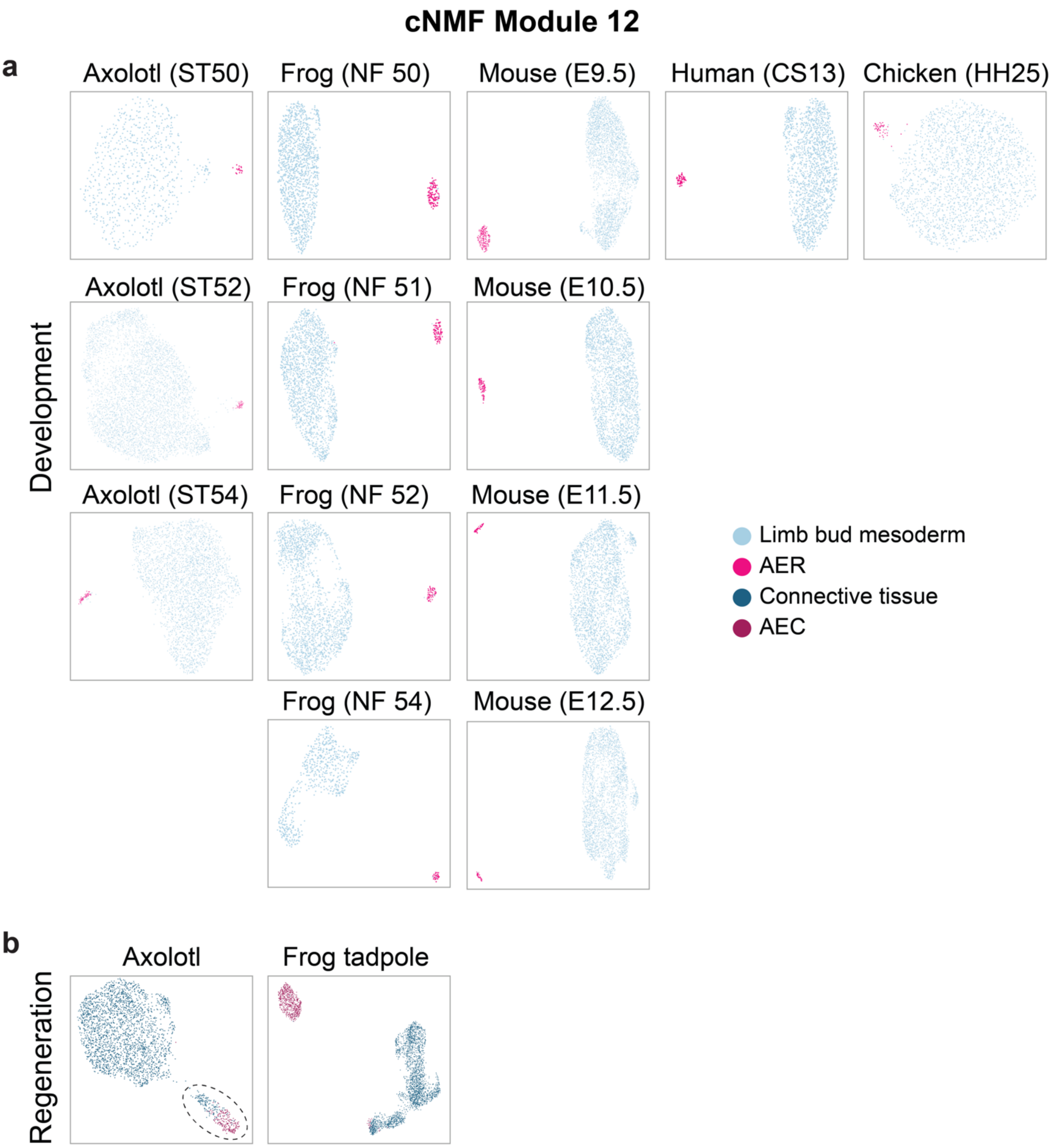

Supplementary Fig. 20 | Clustering based on cNMF module 12 for individual datasets. UMAP visualization of the clustering of indicated datasets of limb development (a) and regeneration (b) based on the top 500 genes from AER-specific cNMF module 12 (Supplementary Fig. 16 and Supplementary Data 4). In development datasets: light blue, limb bud mesoderm cells; pink, AER cells. In regeneration datasets: dark blue, CT cells; dark pink, AEC cells. Connective tissue cells gathered with the AEC population are highlighted with a dashed line.

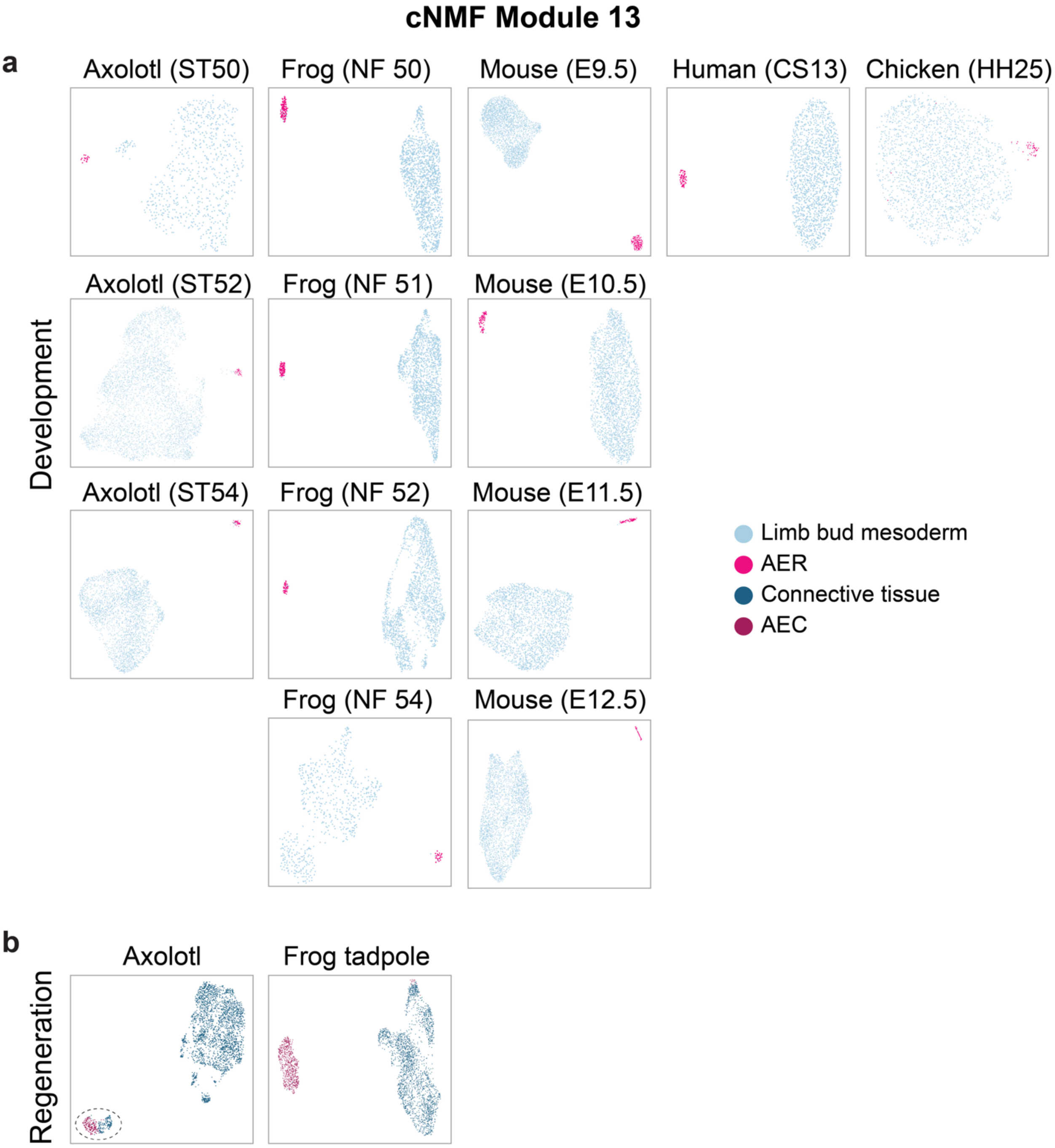

Supplementary Fig. 21 | Clustering based on cNMF module 13 for individual datasets. UMAP visualization of the clustering of indicated datasets of limb development (a) and regeneration (b) based on the top 500 genes from AER-specific cNMF module 13 (Supplementary Fig. 16 and Supplementary Data 4). In development datasets: light blue, limb bud mesoderm cells; pink, AER cells. In regeneration datasets: dark blue, CT cells; dark pink, AEC cells. Connective tissue cells gathered with the AEC population are highlighted with a dashed line.

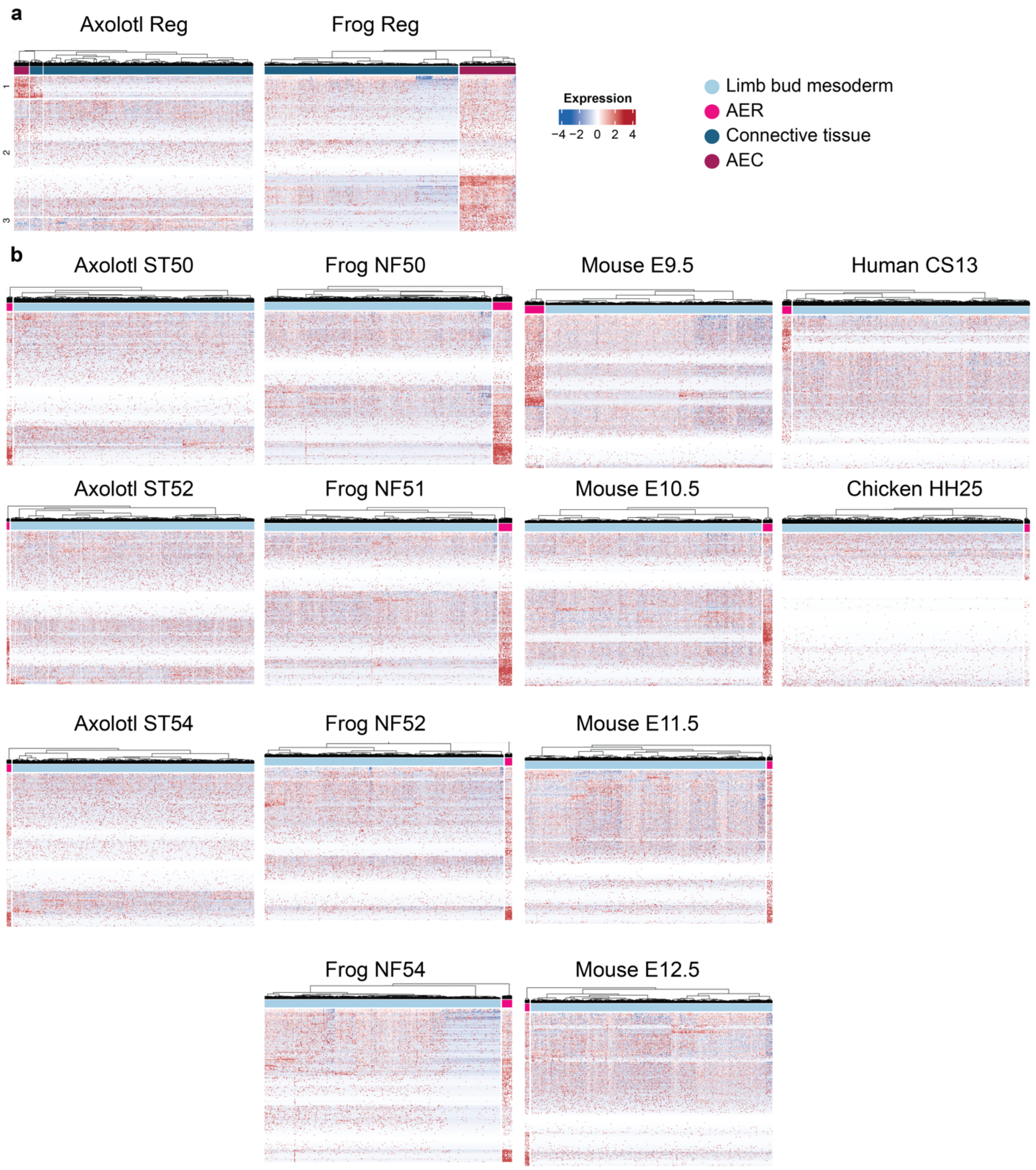

Supplementary Fig. 22 | AER gene expression profile in AER, AEC, limb bud mesoderm, and connective tissue cells during limb development and regeneration.

Heatmaps showing the expression profile of 500 AER DEGs in regenerating limbs (a) and developing limbs (b). For axolotl regenerating limbs (Axolotl Reg), genes were grouped into three by the K-means algorithm, and the first group is shown in Fig. 4d. Please note that part of the axolotl regeneration dataset is also presented in Fig. 4f. Colored squares above the heatmap represent cells from indicated populations: light blue, limb bud mesoderm cells in the development dataset; light pink, AER cells in the development dataset; dark blue, connective tissue cells in the regeneration dataset; dark pink, AEC cells in the regeneration dataset.

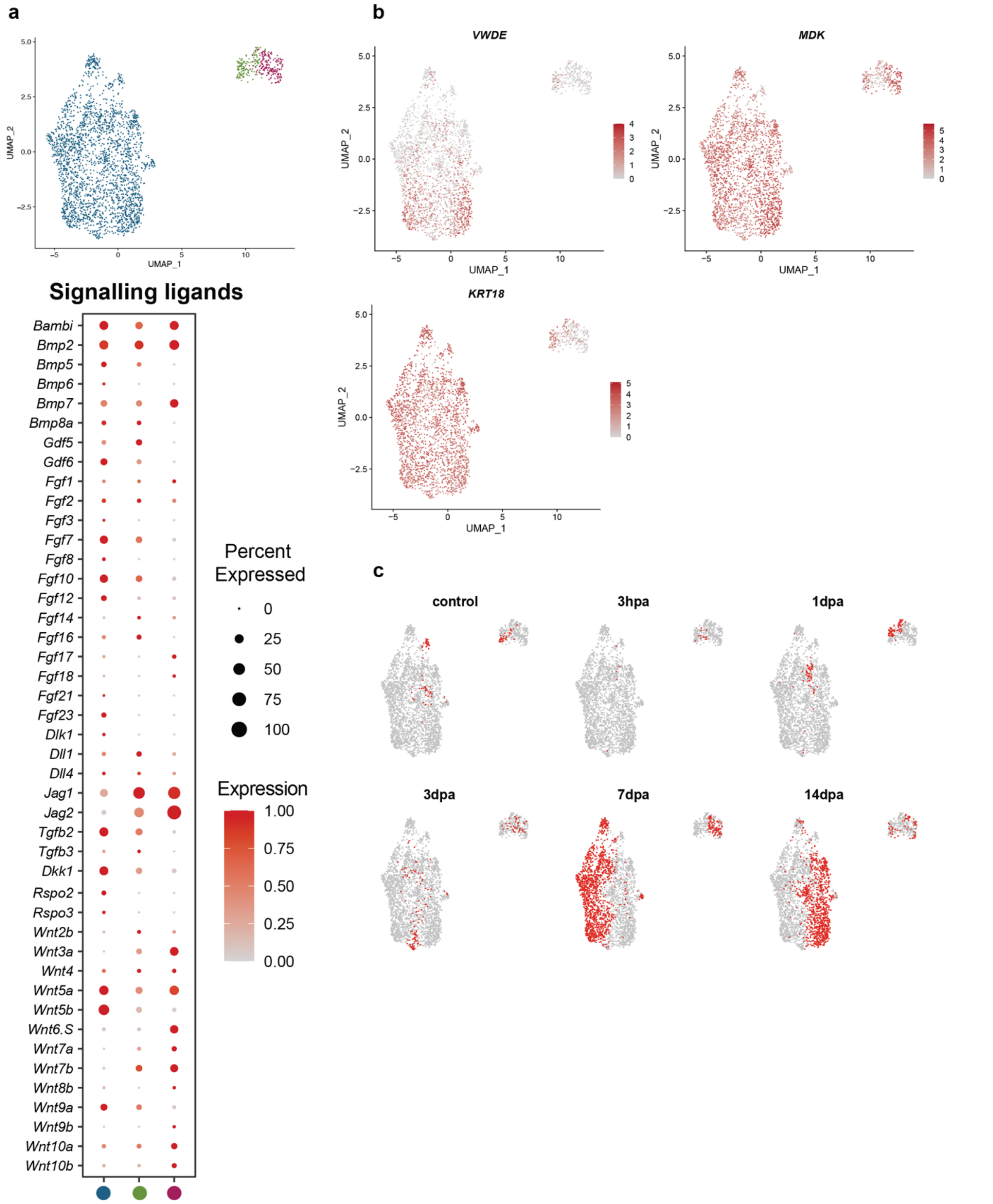

Supplementary Fig. 23 | Mesodermal cells showing AER transcriptional program are present in intact limbs and express signaling ligands.

a) (Top) UMAP plot from Fig. 4c and Supplementary Fig. 18b axolotl regeneration dataset is used to distinguish mesodermal cells showing part of the AER program and labeled by green. The rest of the connective tissue is labeled dark blue, and AEC is labeled dark pink. (Bottom) Dotplot showing signaling ligands expressions in the populations distinguished in Supplementary Fig. 23a. The dot color indicates the mean expression that was normalized to the max of each cell type and to the max of each gene; the dot size represents the percentage of cells with non-zero expression.

b) The expression profile of previously reported regeneration-associated genes (*18*–*20*) in the UMAP plot of clustering in Fig 4c axolotl regeneration dataset.

c) Sample contribution to UMAP plot of re-clustered regeneration dataset is visualized. Red dots indicate cells from the selected sample; gray dots indicate all the other cells.
