# Supplementary material for: Multi-species atlas resolves an axolotl limb development and regeneration paradox
Source: Nat Commun. 2023 Oct 10;14:6346. doi: 10.1038/s41467-023-41944-w (PMC10564727; doi:10.1038/s41467-023-41944-w)
Supplement: Supplementary file 3 — Description of Additional Supplementary Files [file 41467_2023_41944_MOESM3_ESM.pdf]

Description of Additional Supplementary Files

Supplementary Data 1: Quality assessment and details of re-analyzed publicly available datasets.

Supplementary Data 2: Gene sets used for signaling ligands

Supplementary Data 3: Differentially expressed gene analysis for the AER and the AEC. Two-sided Wilcoxon rank sum test was performed. P values were corrected with the Benjamini-Hochberg method.

Supplementary Data 4: Differentially expressed multi-species AER cluster gene set, and AER-specific cNMF module gene sets.
