# Supplementary material for: Multi-species atlas resolves an axolotl limb development and regeneration paradox
Source: Nat Commun. 2023 Oct 10;14:6346. doi: 10.1038/s41467-023-41944-w (PMC10564727; doi:10.1038/s41467-023-41944-w)
Supplement: Supplementary file 8 — Reporting Summary [file 41467_2023_41944_MOESM8_ESM.pdf]

nature portfolio

Corresponding author(s): Tatiana Sandoval-Guzmán and Can Aztekin

Last updated by author(s): Sep 15, 2023

Reporting Summary

Nature Portfolio wishes to improve the reproducibility of the work that we publish. This form provides structure for consistency and transparency in reporting. For further information on Nature Portfolio policies, see our Editorial Policies and the Editorial Policy Checklist.

Statistics

For all statistical analyses, confirm that the following items are present in the figure legend, table legend, main text, or Methods section.

n/a	Confirmed	
☐	☒	The exact sample size (*n*) for each experimental group/condition, given as a discrete number and unit of measurement
☐	☒	A statement on whether measurements were taken from distinct samples or whether the same sample was measured repeatedly
☐	☒	The statistical test(s) used AND whether they are one- or two-sided<br>*Only common tests should be described solely by name; describe more complex techniques in the Methods section.*
☒	☐	A description of all covariates tested
☐	☒	A description of any assumptions or corrections, such as tests of normality and adjustment for multiple comparisons
☐	☒	A full description of the statistical parameters including central tendency (e.g. means) or other basic estimates (e.g. regression coefficient) AND variation (e.g. standard deviation) or associated estimates of uncertainty (e.g. confidence intervals)
☐	☒	For null hypothesis testing, the test statistic (e.g. *F*, *t*, *r*) with confidence intervals, effect sizes, degrees of freedom and *P* value noted<br>*Give P values as exact values whenever suitable.*
☒	☐	For Bayesian analysis, information on the choice of priors and Markov chain Monte Carlo settings
☒	☐	For hierarchical and complex designs, identification of the appropriate level for tests and full reporting of outcomes
☒	☐	Estimates of effect sizes (e.g. Cohen's *d*, Pearson's *r*), indicating how they were calculated

Our web collection on statistics for biologists contains articles on many of the points above.

Software and code

Policy information about availability of computer code

Data collection	sratoolkit(v2.10.9)
Data analysis	Open source software including CellRanger (v6.1.1.1), kallisto|bustools(v0.27.2), SAMap (v1.0.12), tblastx (v2.9.0), SCANPY(1.9.1), kallisto (0.48.0), bustools (0.41.0), SpaceRanger (v2.0.0), cNMF(v1.4), R packages including DropletUtils(v1.12.3), CellChat (v1.1.3), Seurat (v4.0.3), cvms (v1.3.8), MetaNeigbor(v1.12.0), AUCell(v1.14.0), clusterProfiler(4.0.5), web-based tool Metascape were used for data analysis. Custom codes are available in https://github.com/AztekinLab/Axolotl_AER_2023.

For manuscripts utilizing custom algorithms or software that are central to the research but not yet described in published literature, software must be made available to editors and reviewers. We strongly encourage code deposition in a community repository (e.g. GitHub). See the Nature Portfolio guidelines for submitting code & software for further information.

Data

Policy information about availability of data

All manuscripts must include a data availability statement. This statement should provide the following information, where applicable:

- Accession codes, unique identifiers, or web links for publicly available datasets
- A description of any restrictions on data availability
- For clinical datasets or third party data, please ensure that the statement adheres to our policy

The axolotl Visium raw data are publicly available on Gene Expression Omnibus (GEO) under the accession code GSE243225. Previously published scRNA-seq data that were re-analysed here are from NCBI GSE143753(CS13 human limb buds), NCBI GSE157329 (CS13 human limb buds), NCBI GSE137335 (E9.5 mouse limb buds),

NCBI GSE158820 (E10.5, E11.5, E12.5 mouse limb buds), NCBI GSE130439 (HH25 chicken limb buds), NCBI GSE165901 (NF 50, NF51, NF52 frog limb buds; Stage 50, 52, 54 axolotl limb buds), ArrayExpress E-MTAB-9104 (NF 54 frog limb buds, 5 days post-amputation NF 52 frog regenerating limbs), NCBI PRJNA589484 (0-14 days post-amputation axolotl regenerating limbs).

Research involving human participants, their data, or biological material

Policy information about studies with human participants or human data. See also policy information about sex, gender (identity/presentation), and sexual orientation and race, ethnicity and racism.

Reporting on sex and gender	No relevant. No human participants involved in this study.
Reporting on race, ethnicity, or other socially relevant groupings	No relevant. No human participants involved in this study.
Population characteristics	No relevant. No human participants involved in this study.
Recruitment	No relevant. No human participants involved in this study.
Ethics oversight	No relevant. No human participants involved in this study.

Note that full information on the approval of the study protocol must also be provided in the manuscript.

Field-specific reporting

Please select the one below that is the best fit for your research. If you are not sure, read the appropriate sections before making your selection.

[x] Life sciences [ ] Behavioural & social sciences [ ] Ecological, evolutionary & environmental sciences

For a reference copy of the document with all sections, see nature.com/documents/nr-reporting-summary-flat.pdf

Life sciences study design

All studies must disclose on these points even when the disclosure is negative.

Sample size	No statistic method was used to estimate sample size. The sample size was based on the number of individuals available.
Data exclusions	Low quality cells were excluded as described in the Methods.
Replication	Replicates for the HCR experiments are reported as follows. Technical replicates (total number of different animals used) are denoted as n, and independent replicates (total number of experiments performed on separate days, and/or with different batches) are denoted as N. For stage 46 axolotls, n=20, N=7 (Dr999); n=16, N=5 (Vwa2); n=6, N=2 (Msx2, Epcam); n=10, N=2 (Fgf7, Fgf18). For stage 50 axolotls, n=5, N=2 (Dr999 and Vwa2). For stage 53 axolotls, n=20, N=7 (Dr999); n=16, N=5 (Vwa2); n=4, N=2 (Msx2, Epcam). For E10.5 mice, n=15, N=4 (Fgf8); n=10, N=3 (Vwa2). For HH22 chicken, n=4, N=2 (FGF8, VWA2). All attempts at replication were successful.
Randomization	Not relevant This study used publicly available datasets and no treatment groups are involved.
Blinding	Investigators were not blinded. Both the data collection and the analysis required an understanding of the nature of the sample being used. Blinding is also not necessary because the results are quantitative and did not require subjective judgment or interpretation.

Reporting for specific materials, systems and methods

We require information from authors about some types of materials, experimental systems and methods used in many studies. Here, indicate whether each material, system or method listed is relevant to your study. If you are not sure if a list item applies to your research, read the appropriate section before selecting a response.

Materials & experimental systems

n/a	Involved in the study	
x		Antibodies
x		Eukaryotic cell lines
x		Palaeontology and archaeology
	x	Animals and other organisms
x		Clinical data
x		Dual use research of concern
x		Plants

Methods

n/a	Involved in the study	
x		ChIP-seq
x		Flow cytometry
x		MRI-based neuroimaging

Animals and other research organisms

Policy information about studies involving animals; ARRIVE guidelines recommended for reporting animal research, and Sex and Gender in Research

Laboratory animals	Axolotl (Ambystoma mexicanum) husbandry was performed in the CRTD axolotl facility using methodology adapted from Khattak et al.61 and according to the European Directive 2010/63/EU, Annex III, Table 9.1. Axolotls were kept in 18-19°C water in a 12 h light/12 h dark cycle and a room temperature of 20-22°C. Animals were housed in individual tanks categorized by a water surface (WS) area and a minimum water height (MWH). Axolotls of a size up to 5 cm SV were maintained in tanks with a WS of 180 cm2 and MWH of 4.5 cm. Axolotls up to 9 cm SV were maintained in tanks with a WS of 448 cm2 and MWH of 8 cm.<br><br>Pregnant CD-1 mice (Mus musculus) were obtained from Charles River Laboratories. The mice were housed in rooms with a regular dark/light cycle and fed a standard rodent diet and water ad libitum.<br><br>Chickens (Gallus gallus) were outbred and chicken eggs were obtained from a local farm (Brüterei Stöckli AG). Eggs were incubated at 38 °C and staged according to the Hamburger-Hamilton staging chart.
Wild animals	This study did not involve wild animals.
Reporting on sex	Animals sex information was not collected.
Field-collected samples	This study did not involve field-collected animals.
Ethics oversight	Axolotls (Ambystoma mexicanum) husbandry and experimental procedures were performed according to the Animal Ethics Committee of the State of Saxony, Germany. All mice and chickens embryo samples were collected in accordance with the Swiss Federal Veterinary Office guidelines and as authorized by the Cantonal Veterinary Office (cantonal animal license no: VD3652c, and national animal license no: 33237). No sex determination was performed by any of the used animal samples in this study.

Note that full information on the approval of the study protocol must also be provided in the manuscript.
